# The long noncoding RNA nHOTAIRM1 is necessary for differentiation and activity of iPSC-derived spinal motor neurons

**DOI:** 10.1038/s41419-023-06196-y

**Published:** 2023-11-14

**Authors:** Paolo Tollis, Erika Vitiello, Francesco Migliaccio, Eleonora D’Ambra, Anna Rocchegiani, Maria Giovanna Garone, Irene Bozzoni, Alessandro Rosa, Annamaria Carissimo, Pietro Laneve, Elisa Caffarelli

**Affiliations:** 1 Department of Biology and Biotechnologies “C. Darwin”, Sapienza University of Rome, Rome, Italy; 2 Center for Life Nano-& Neuro-Science, Fondazione Istituto Italiano di Tecnologia, Rome, Italy; 3 Center for Human Technology, Fondazione Istituto Italiano di Tecnologia, Genoa, Italy; 4 Department of Electrical Engineering and Information Technology, University Federico II, Naples, Italy; 5 Institute for Applied Mathematics “Mauro Picone”, CNR, Naples, Italy; 6 The Novo Nordisk Foundation Center for Stem Cell Medicine, reNEW Melbourne, Murdoch Children’s Research Institute, Parkville, VIC 3052 Australia; 7 Institute of Molecular Biology and Pathology, Rome, CNR Italy; 8 Present Address: Division of Cell and Developmental Biology, Center for Anatomy and Cell Biology, Medical University of Vienna, Vienna, Austria; 9 Present Address: Stem Cell Biology Department, Murdoch Children’s Research Institute, Parkville, VIC 3052 Australia

**Keywords:** Long non-coding RNAs, Differentiation

## Abstract

The mammalian nervous system is made up of an extraordinary array of diverse cells that form intricate functional connections. The programs underlying cell lineage specification, identity and function of the neuronal subtypes are managed by regulatory proteins and RNAs, which coordinate the succession of steps in a stereotyped temporal order. In the central nervous system (CNS), motor neurons (MNs) are responsible for controlling essential functions such as movement, breathing, and swallowing by integrating signal transmission from the cortex, brainstem, and spinal cord (SC) towards peripheral muscles. A prime role in guiding the progression of progenitor cells towards the MN fate has been largely attributed to protein factors. More recently, the relevance of a class of regulatory RNAs abundantly expressed in the CNS - the long noncoding RNAs (lncRNAs) - has emerged overwhelmingly. LncRNA-driven gene expression control is key to regulating any step of MN differentiation and function, and its derangement profoundly impacts neuronal pathophysiology. Here, we uncover a novel function for the neuronal isoform of *HOTAIRM1* (*nHOTAIRM1*), a lncRNA specifically expressed in the SC. Using a model system that recapitulates spinal MN (spMN) differentiation, we show that *nHOTAIRM1* intervenes in the binary cell fate decision between MNs and interneurons, acting as a pro-MN factor. Furthermore, human iPSC-derived spMNs without *nHOTAIRM1* display altered neurite outgrowth, with a significant reduction of both branch and junction numbers. Finally, the expression of genes essential for synaptic connectivity and neurotransmission is also profoundly impaired when *nHOTAIRM1* is absent in spMNs. Mechanistically, *nHOTAIRM1* establishes both direct and indirect interactions with a number of target genes in the cytoplasm, being a novel post-transcriptional regulator of MN biology. Overall, our results indicate that the lncRNA *nHOTAIRM1* is essential for the specification of MN identity and the acquisition of proper morphology and synaptic activity of post-mitotic MNs.

## Introduction

Motor neurons (MNs) generate complex networks that propagate nerve impulses from the central nervous system (CNS) to peripheral tissues, where they are translated into muscle contraction [1, 2]. Special attention has recently been trained to MN development and function, since these cells are particularly vulnerable in severe degenerative diseases [3]. In mammals, upper MNs establish glutamatergic connections with lower cholinergic MNs, conveying the information to the target muscles. Among lower MNs, spinal MNs (spMNs) represent the ultimate link of the neuronal circuit to skeletal muscles.

MN lineage determination is dictated by signaling pathways coordinating the assembly of cell type-specific transcription factors (TFs), which trigger MN-specific gene programs, eventually suppressing alternative differentiation fates. The TFs OLIG2 and HB9 are paradigmatic for inducing spMN specification while inhibiting the interneuron (IN) identity [4, 5]. A class of regulatory noncoding RNAs that also plays a central role in MN differentiation are the long noncoding RNAs (lncRNAs), that share several features with messenger RNAs (mRNAs) but are not translated into proteins [6]. They are implicated in most cellular events for their function as transcriptional and post-transcriptional regulators of gene expression, conjugated with their capability to simultaneously interact with nucleic acids and proteins [7]. These distinctive traits rely on their ability to fold into multiple functional domains, achieving a modular organization through which they bind and coordinate the activity of different factors. This makes lncRNAs very versatile molecules endowed with high specificity of action.

Their pervasive expression in the CNS [8], where they are tightly regulated in space and time, strongly argues for their implication in neurodevelopmental processes and in human brain evolution [9, 10]. Moreover, the profound impact of lncRNA deregulation on MN pathophysiology is diagnostic of their involvement in orchestrating cell fate decision, cell identity and function of this neuronal subtype [6]. Despite this, very few lncRNAs have been associated with MN development and activity [11], with a main function as transcriptional regulators [6, 7].

We previously characterized the neuronal isoform of *HOTAIRM1*, which we referred to as *nHOTAIRM1*, as a lncRNA upregulated during in vitro neuronal differentiation of SH-SY5Y cells, considered as equivalent to neuronal precursors [12]. Physiologically, among the brain tissues, *nHOTAIRM1* is exclusively expressed in the spinal cord (SC). In line with this observation, we found that it accumulates in MN-enriched ventral SC lineages, differentiated from human induced pluripotent stem cells (iPSCs) [12]. This evidence prompted us to explore whether *nHOTAIRM1* might impact MN generation and function, to finally realize how its deregulation may affect MN life cycle. Here, we applied a genome editing-based loss-of-function approach to a model system that recapitulates spMN differentiation and identified key *nHOTAIRM1* target genes implicated in MN development and function. We show that *nHOTAIRM1* is critical in multiple aspects of MN physiology, from their formation, at the expenses of INs, to the achievement of their correct morphology and activity.

## Results

### *nHOTAIRM1* is induced in a model of spMN differentiation

**Fig. 1:**
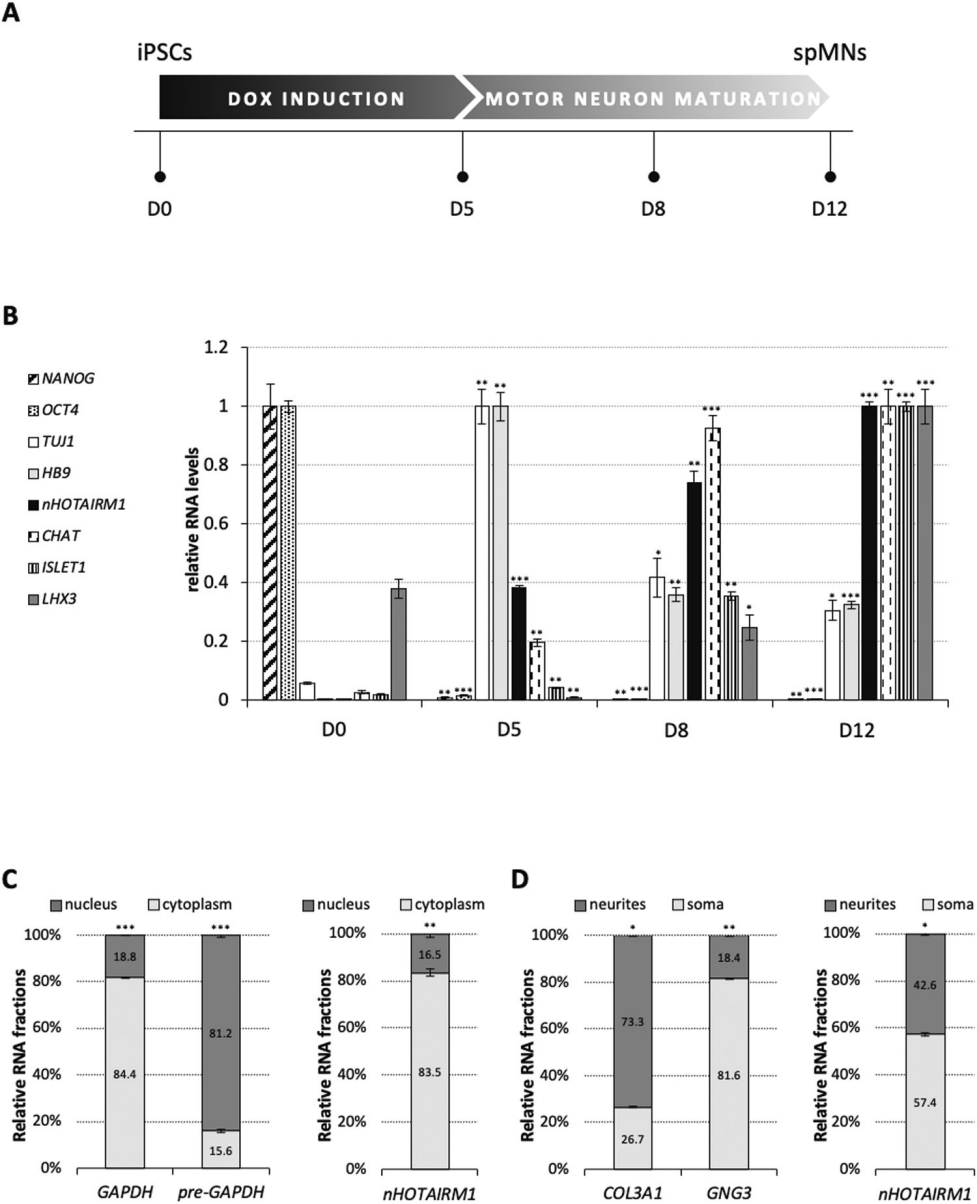
*nHOTAIRM1* expression profile along spMN differentiation and its distribution in post-mitotic spMNs. **A** Schematic representation of a 12-day protocol for differentiating functional spMNs from iPSCs. Cells were induced for 5 days by doxycycline treatment (DOX induction, D0-D5) for the expression of programming TFs and then cultured for further 7 days (D12) for spMN maturation. **B** Expression profiling of differentiation markers and *nHOTAIRM1* along iPSC conversion to spMNs. qRT-PCR analysis of markers for: pluripotency (*NANOG, OCT4*), MN progenitors (*TUJ1*, *HB9*), MN specification (*ISLET1*), spinal identity (*LHX3*), electrophysiological activity (*ChAT*). *nHOTAIRM1* expression is indicated by black bars. Differentiation days are indicated on the x-axis. Data (means ± SEM) are expressed as arbitrary units, relative to *ATP5O* mRNA levels, as internal control. For each target, the peak of expression was set as 1. D0 was considered as the reference sample for statistical tests. **C** qRT-PCR analysis of *nHOTAIRM1* nucleus/cytoplasm localization in spMNs (D12). Left panel: the efficiency of nucleus/cytoplasm fractionation in spMNs was assessed by evaluating *pre*-*GAPDH* and *GAPDH* RNA levels, respectively. Normalization was performed on total RNA. Data (means ± SEM) are expressed as percentage of total *GAPDH* or *pre-GAPDH* levels. Right panel: *nHOTAIRM1* distribution in the nucleus (dark gray) and cytoplasm (light gray) of spMNs. Normalization was performed on total RNA. Data (means ± SEM) are expressed as percentage of total *nHOTAIRM1* levels. **D** qRT-PCR analysis of *nHOTAIRM1* distribution in the soma and the neurites of spMNs (D12). Left panel: efficiency of soma/neurite separation from spMNs was assessed by evaluating the levels of *GNG3* and *COL3A1* mRNAs, respectively. Normalization was performed on total RNA. Data (means ± SEM) are expressed as percentage of total *GNG3* and *COL3A1* levels. Right panel: *nHOTAIRM1* distribution in the neuritic (dark gray) and somatic (light gray) compartments. Normalization was performed on total RNA. Data (means ± SEM) are expressed as percentage of total *nHOTAIRM1* levels. In all the panels, statistics is as follows: *N* = 3 biological replicates; *P ≤ 0.05, **P ≤ 0.01, ***P ≤ 0.001, two-tailed Student’s *t* test.

iPSCs were differentiated into functional spMNs, following a protocol based on lineage-specific inducible transcriptional programming modules (NIL) [13, 14]. The progression of differentiation induced by doxycycline (Dox) treatment (Fig. 1A) was monitored via qRT-PCR analysis of specific cellular markers (Fig. 1B). *NANOG* and *OCT4* were used as iPSC pluripotency markers (D0), whereas the conversion of iPSCs into MN progenitors (MNPs, D5) was signed by the expression of the pan-neuronal marker *TUJ1* and of the early marker of cholinergic MNs *MNX1* (referred as *HB9* throughout the text) [15]. At this stage, MNPs were dissociated and re-plated for further maturation. Three days later (D8), cells displayed consistent expression of *ChAT*, required for the synthesis of the neurotransmitter acetylcholine [16]. On day 12 (D12), a marked induction was observed for *LHX3* and *ISLET1*, whose coordinated expression directs cells to become spMNs [17]. At this stage, the cell population is almost exclusively composed of post-mitotic spMNs displaying the properties of functional MNs [14]. In this differentiation set, we profiled *nHOTAIRM1* expression (Fig. 1B). Almost undetectable in iPSCs, it was gradually induced in MNPs, reaching its peak of expression in spMNs (D12). This marked increase in expression suggests the involvement of *nHOTAIRM1* in spMN programming, whereas its high expression levels in post-mitotic MNs also indicate a potential role in spMN activity.

### *nHOTAIRM1* is mainly cytoplasmic and localized in the soma and the neurites of spMNs

To unravel the function of *nHOTAIRM1* in spMNs, we analyzed its subcellular localization. Biochemical fractionation and expression analysis in spMNs (D12), using *GAPDH* and *pre*-*GAPDH* as controls (Fig. 1C, left panel), revealed that *nHOTAIRM1* is predominantly cytoplasmic (about 83%, Fig. 1C, right panel). This suggests its potential role as a post-transcriptional regulator of gene expression. We next deepened the distribution of *nHOTAIRM1* in spMN cell bodies (somata) and neurites. Upon soma/neurite separation, assessed by checking *GNG3* and *COL3A1* localizations, respectively [18, 19] (Fig. 1D left panel), the relative enrichment of the lncRNA was quantified (Fig. 1D, right panel), revealing its occurrence at comparable amounts in both districts, with the cell bodies being also contributed by the nuclear counterpart.

**Fig. 2:**
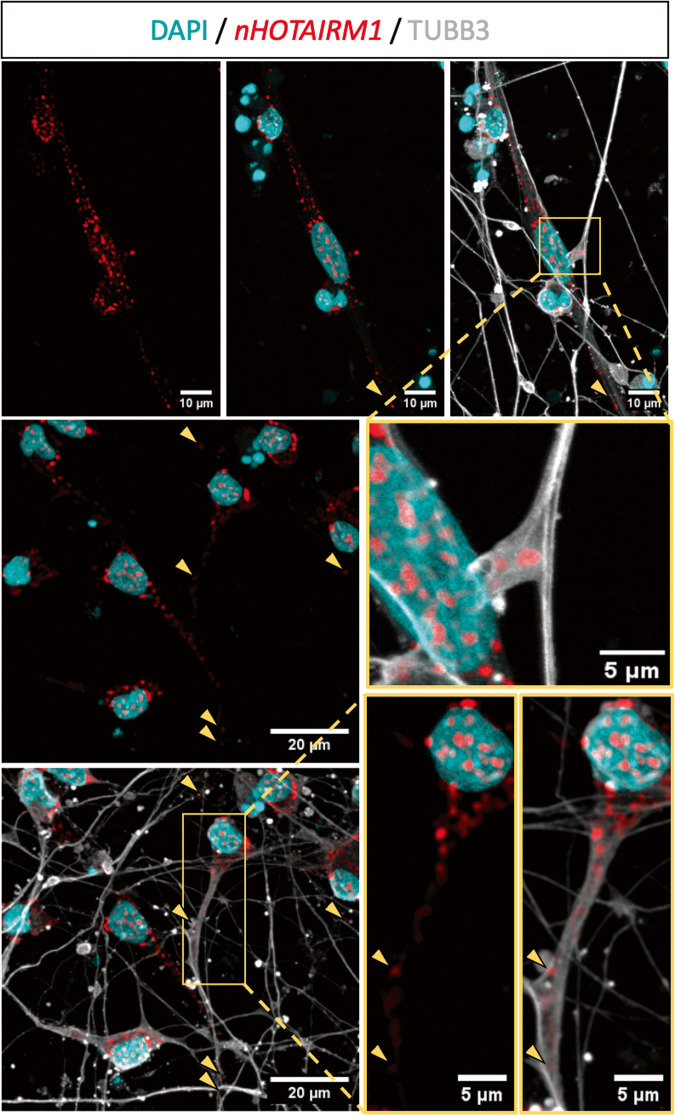
Visualization of *nHOTAIRM1* subcellular localization in post-mitotic spMNs. RNA FISH of *nHOTARIM1* (red spots) combined with TUBB3 IF (gray) in iPSC-derived spMNs (D12). Nuclei are counterstained in cyan. Square inserts are digitally magnified in yellow-bordered panels. Yellow arrowheads point to *nHOTAIRM1* spots in distal axonal segments. Scale bar units are indicated in each panel.

This distribution was confirmed by imaging assays combining RNA fluorescence in situ hybridization (RNA FISH) with immunofluorescence (IF). Figure 2 shows that *nHOTAIRM1* (indicated by red spots) is enriched in the soma, but also accumulates in the neurites, as well as in distal axonal segments (yellow arrowheads). These findings imply potential roles for *nHOTAIRM1* in diverse functions such as neurite outgrowth, axonal transport, local translation of axon-resident mRNAs or synaptic activity.

### Identification of *nHOTAIRM1* target genes through transcriptome analysis

**Fig. 3:**
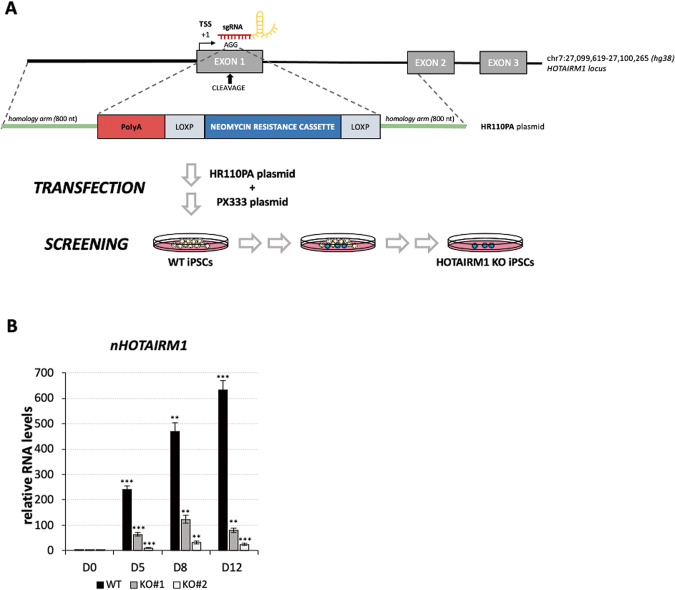
*nHOTAIRM1* functional KO. **A** Schematic representation of *HOTAIRM1* gene editing experiment. Two sgRNAs (carried by the plasmid PX333) were designed to target exon 1 of *HOTAIRM1 locus*. The polyA signal, the selection cassette, and the homology arms for homology-directed repair through DNA recombination were carried by the HR110PA plasmid. Cell manipulation is schematized below. **B** qRT-PCR analysis of *nHOTAIRM1* expression in wild type (WT) and *nHOTAIRM1* KO (KO#1 and KO#2) spMNs (D12). Data (means ± SEM) are expressed in arbitrary units, relative to *ATP5O* mRNA levels, as internal control. *N* = 3 biological replicates; *P ≤ 0.05, **P ≤ 0.01, ***P ≤ 0.001, two-tailed Student’s *t* test, referred to D0 as the reference sample for statistical tests.

To decode *nHOTAIRM1* function, its target genes were identified through transcriptomic analysis performed in wild-type (WT) and *HOTAIRM1* knockout (KO) spMNs (D12). The KO iPSC lines were obtained using CRISPR/Cas9 genome editing by blocking *HOTAIRM1* gene transcription through a premature poly-A signal [20], inserted at the 5’ region of its first exon, downstream to the annotated transcription start site peaks reported in Zenbu (https://fantom.gsc.riken.jp/zenbu/) (Fig. 3A). Two independent *HOTAIRM1* KO homozygous iPSC clones (KO#1 and KO#2) were generated. Figure 3B shows that the induction of the lncRNA is drastically reduced along the entire differentiation process of both KO clones, achieving a reduction by about 87% and 96% in differentiated KO#1 and KO#2 spMNs (D12), respectively.

Data mining of RNA-Seq analyses, performed on three independent biological replicates of WT and KO#2 spMNs, showed that both samples clustered homogeneously and produced read counts for many genes (Supplementary Fig. 1A, B). We detected 16,504 genes expressed in at least one sample (Dataset 1) and, among them, those having |logFC | > 1 and adjusted *p*-value < 0.05 were considered as significantly differentially expressed. Applying these filters, we found 1,887 upregulated and 654 downregulated genes (Dataset 1) in KO spMNs, considered as *bona fide nHOTAIRM1* targets (Supplementary Fig. 1C–E). Gene ontology over-representation analysis was performed on the lists of differentially expressed genes using WebGestalt R tool (http://www.webgestalt.org/). From this test, only the gene sets with FDR < 0.05 were considered significantly enriched. Concerning the upregulated genes, the analysis of “Biological Process”, “Cellular Component” and “Molecular Function” domains revealed highly heterogeneous ontological annotations, not strictly related to MN biology (Supplementary Figs. 2–4, panels A). Similar indications derived from querying the KEGG and the REACTOME pathway databases (Supplementary Figs. 5,6, panels A). When applied to downregulated genes, all these computational inspections indicated, instead, that they were enriched for neuronal functions (Supplementary Figs. 2–6, panels B). Of note, the “Biological Process” ontology showed annotations clearly associated to MN (i) differentiation, (ii) morphology, and (iii) activity (Dataset 1 and Supplementary Fig. 2B), which guided the subsequent analyses.

To first validate the RNA-Seq analysis, subsets of ten upregulated and ten downregulated genes were analyzed by qRT-PCR in WT and KO spMNs. Candidates with an adjusted *p*-value < 0.05 and having different significance values ranging from logFC −2.72 to logFC 7.65 were selected. Notably, all of them followed the expression trend highlighted in the RNA-Seq analysis (Supplementary Fig. 7A, B).

Subsequent functional studies focused on three main groups of genes belonging to seven downregulated biological processes, implicated in fundamental aspects of MN biology. They included over-represented genes engaged in: (i) MN differentiation (“cell differentiation in spinal cord” and “CNS neuronal differentiation”); (ii) morphology (“neuron projection guidance”, “axonogenesis”, and “axon guidance”), and (iii) activity (“synapse organization”, and “modulation of chemical synaptic transmission”).

### *nHOTAIRM1* regulates binary fate decision between MNs and INs

**Fig. 4:**
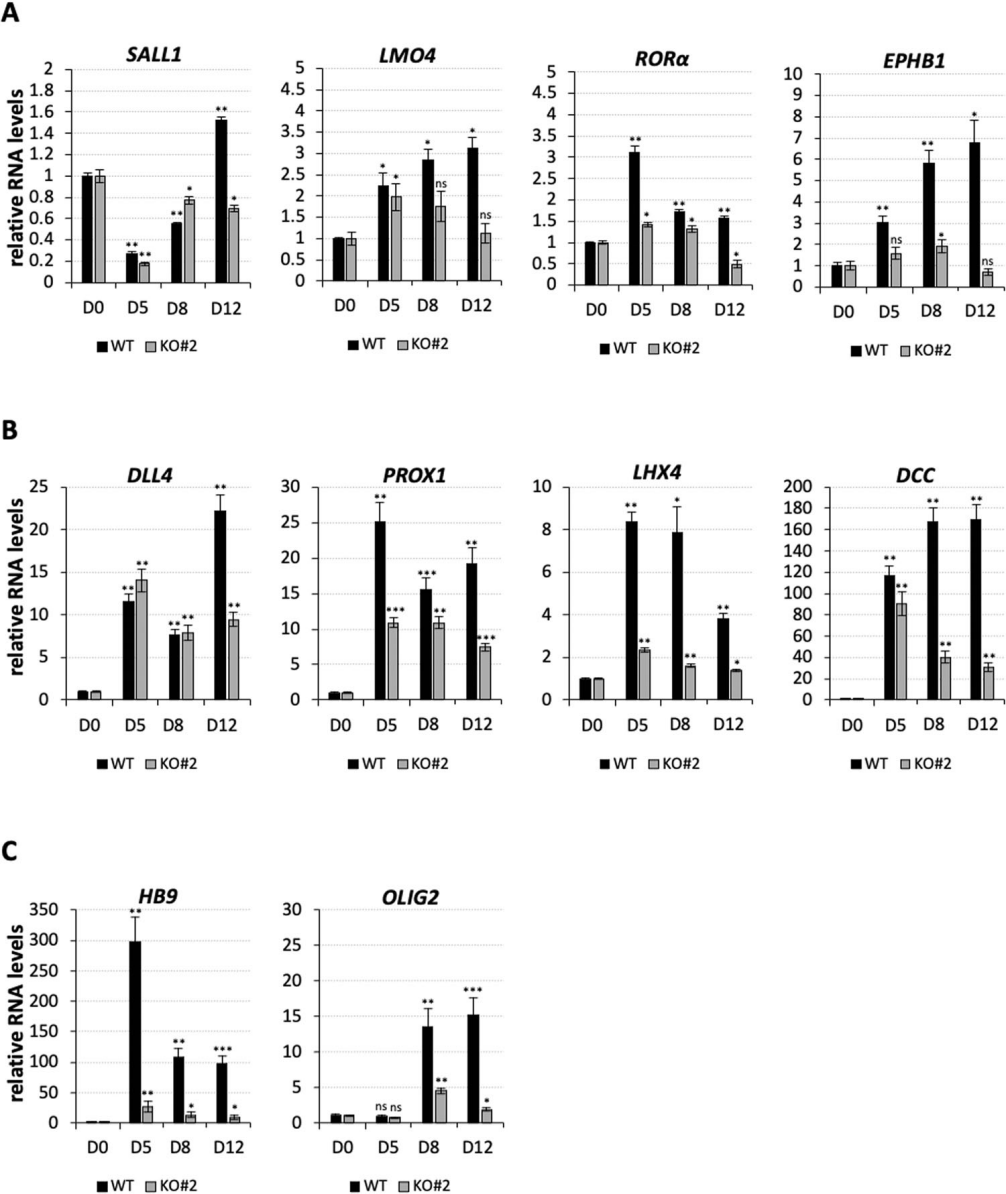
Expression analysis of genes related to neuronal differentiation along the conversion of WT and *nHOTAIRM1* KO iPSCs into post-mitotic spMNs. **A**, **B** Expression profile, by qRT-PCR analysis, of genes (indicated above each panel) implicated in neuronal development (**A**) and linked to SC or MN development (**B**), along the differentiation (from D0 to D12, x-axis) of WT or KO for *nHOTAIRM1* spMNs (KO#2 clone). **C** Expression profile, by qRT-PCR analysis of *HB9* and *OLIG2* genes along the differentiation (from D0 to D12, x-axis) of WT or KO for *nHOTAIRM1* spMNs (KO#2 clone). Data (means ± SEM) are expressed as arbitrary units in all the panels, relative to *ATP5O* mRNA levels, as internal control. For each target and each cell line, the expression at D0 was set as 1. *N* = 3 biological replicates; *P ≤ 0.05, **P ≤ 0.01, ***P ≤ 0.001, two-tailed Student’s *t*-test, referred to D0 as the reference sample for statistical tests.

To explore the involvement of *nHOTAIRM1* in neuronal cell fate decision, the expression profile of target genes related to differentiation was compared between WT and KO iPSCs along their conversion into spMNs (Fig. 4). We confirmed in KO cells the altered expression pattern of genes implicated in neuronal development, such as the TF and cofactor *SALL1* [ref. [21]] and *LMO4* [ref. [22]], expressed in the developing cerebral cortex, the nuclear receptor *RORa*, which identifies excitatory neurons in the CNS [23] and the transmembrane receptor *EPHB1*, important for the regulation of contact-dependent cell-to-cell interactions [24] (Fig. 4A). We also validated the deregulation of genes linked to SC or MN development (Fig. 4B), such as *DLL4*, with a role in neuronal subtype specification in the SC [25], *PROX1*, a regulator of ventral SC patterning [5], *LHX4*, required for ventral MN differentiation [26] and *DCC*, associated with MN migration [27]. The expression profiles of these genes were altered along the formation of KO spMNs from iPSCs, with a range of decrease spanning from 54% to 90% at D12. This suggests that *nHOTAIRM1* is required for (motor) neuron development.

**Fig. 5:**
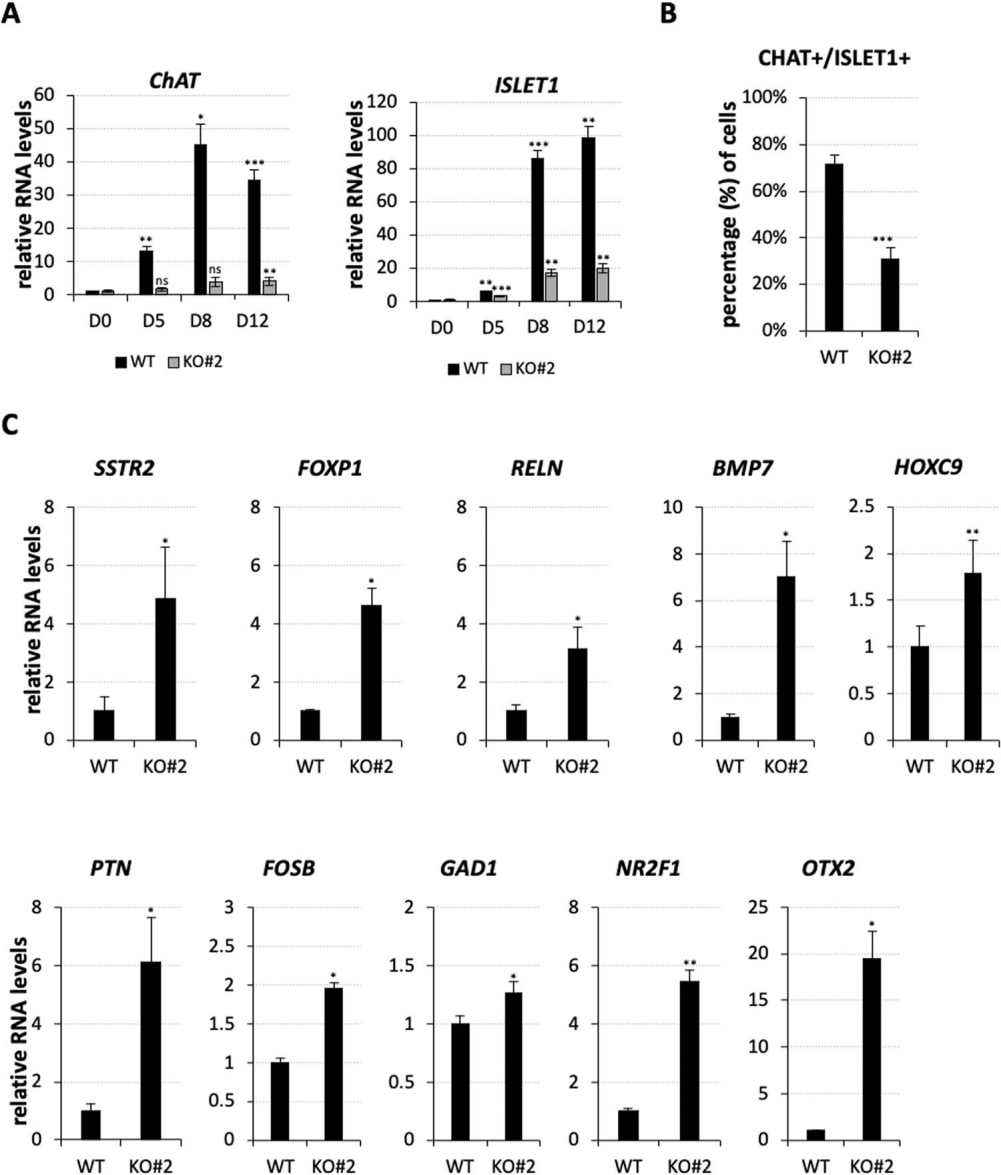
Analysis of MN and HB9-dependent IN genes upon *nHOTAIRM1* KO. **A** Expression profile, by qRT-PCR analysis, of *ChAT* and *ISLET1* genes along the differentiation (from D0 to D12, x-axis) of WT or KO for *nHOTAIRM1* spMNs (KO#2 clone). Data (means ± SEM) are expressed as arbitrary units, relative to *ATP5O* mRNA levels, as internal control. Expression levels at D0 were set as 1. *N* = 3 biological replicates; *P ≤ 0.05, **P ≤ 0.01, ***P ≤ 0.001, two-tailed Student’s *t*-test, referred to D0 as the reference sample for statistical tests. **B** Quantification by immunostaining of CHAT + /ISLET1+ cells, expressed as percentage of the total number of cells, in WT and *nHOTAIRM1* KO (KO#2) spMNs (D12). *N* = 3 groups of 60 cells randomly selected from 9 different fields, both for WT and KO, were analyzed. ***P ≤ 0.001. **C** qRT-PCR validation, in WT and *nHOTAIRM1* KO (KO#2 clone) spMNs (D12) of the expression of HB9-dependent IN genes (indicated above each panel) significantly upregulated in *HB9* KO MNs and *nHOTAIRM1* KO spMNs compared to WT spMNs. Data (means ± SEM) are expressed in arbitrary units, relative to *ATP5O* mRNA levels, as internal control. For each target, the expression in WT spMNs was set as 1. *N* = 3 biological replicates; *P ≤ 0.05, **P ≤ 0.01, ***P ≤ 0.001, two-tailed Student’s *t* test.

A consistent downregulation along the differentiation of KO spMNs from iPSCs was also appreciated for *HB9* and *OLIG2* (Fig. 4C), two genes crucially implicated in acquiring alternative neuronal fates. The *HB9* gene, a selective marker of MNs in the developing SC [4, 28], is critical for specifying MN identity and repressing the IN character. Similarly, its regulator OLIG2, primes MNPs to become MNs [29] and inhibits IN identity [5]. On this basis, we wondered whether *nHOTAIRM1* could positively control the generation of MNs at the expense of INs. To answer this question, we analyzed the expression of the cholinergic neuron marker *ChAT* [30] and the major MN marker *ISLET1* [ref. [4]] during the conversion of iPSCs into spMNs. We found their expression levels drastically reduced along the differentiation of KO iPSCs (Fig. 5A), highlighting the relevance of *nHOTAIRM1* in spMN generation. This finding was corroborated by IF analysis (Fig. 5B and Supplementary Fig. 8), in which we observed a significant decrease by about 42% in the number of CHAT + /ISLET1 + MN cells upon *nHOTAIRM1* depletion. In this scenario, where the lack of *nHOTAIRM1* impairs spMN differentiation, we asked whether an alternative cell fate was undertaken. Given that *nHOTAIRM1* regulates the expression of *HB9*, a molecular switch between MN and IN cell fate, we wondered whether the lack of the lncRNA could partially mimic the absence of *HB9*.

To verify this, we first intersected the lists of significantly upregulated genes in *nHOTAIRM1* KO spMNs and *HB9*-null mutant mouse-induced MNs [31]. We found a signature of 10 shared genes expressed in INs that we validated in KO spMNs (D12) via qRT-PCR (Fig. 5C). Collectively, these results indicate that *nHOTAIRM1*, through the control of *HB9* expression, regulates the binary fate decision between INs and MNs, to the latter’s advantage. This evidence was definitively confirmed by the analysis of specific IN marker genes, namely *CALB2*, *SST*, *ETV1*, *LHX1*, *LAMP5*, *PVALB*, and *VIP*, that we found upregulated upon *nHOTAIRM1* depletion (Supplementary Fig. 9A).

### *nHOTAIRM1* is necessary for the correct MN neurite outgrowth

**Fig. 6:**
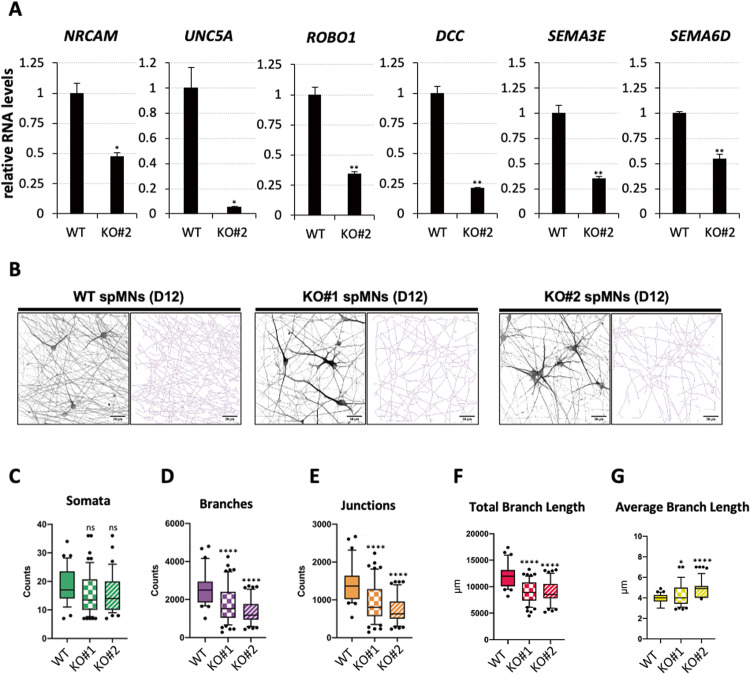
Analysis of the neurite network in WT and *nHOTAIRM1* KO post-mitotic spMNs. **A** qRT-PCR analysis of genes involved in neurite outgrowth and branching (indicated above each panel) in WT and *nHOTAIRM1* KO (KO#2 clone) spMNs (D12). Data (means ± SEM) are expressed in arbitrary units, relative to *ATP5O* mRNA levels, as internal control. For each target, the expression in WT spMNs was set as 1. *N* = 3 biological replicates; *P ≤ 0.05, **P ≤ 0.01, ***P ≤ 0.001, two-tailed Student’s *t* test. **B** Representative acquisition and skeletonization of neurite networks in WT and *nHOTAIRM1* KO (KO#1 and KO#2 clones) spMNs (D12). Scale bar units are indicated in each panel. **C**–**G** Number of somata (**C**), number of branches (**D**), number of junctions (**E**), total branch length (**F**), and average branch length (**G**) per acquisition in WT and *nHOTAIRM1* KO (KO#1 and KO#2 clones) spMNs (D12). Horizontal lines in boxplots indicate median values; boxes extend from the 25th to the 75th percentile of each group’s distribution of values; black dots represent outliers. Boxplots represent the value distribution from *N* = 3 independent biological replicates. In all, 10–20 acquisitions with a size of 212.3 μm × 212.3 μm have been analyzed for each replicate. *P ≤ 0.05, ****P ≤ 0.0001 correspond to one-way ANOVA multiple comparison test.

Neurite outgrowth depends on the activity of several genes promoting the formation of functional neuronal connections [32]. Interestingly, comparative transcriptome analysis revealed a list of genes critically involved in neurite outgrowth which were downregulated in KO spMNs. We focused on six of them, namely *NrCAM*, *UNC5A*, *ROBO1*, *DCC*, *SEMA3E* and *SEMA6D*, found enriched in three ontological gene categories, i.e. “neuron projection guidance”, “axon guidance”, and “axonogenesis”. In particular, the expression of *NrCAM*, crucially implicated in neurite outgrowth activity in vitro [33], and of *UNC5A*, involved in axonal pathfinding [34], declined by about 53% and 95%, respectively (Fig. 6A). The expression of the receptor *ROBO1*, engaged in axon guidance through the interaction with SLIT ligands [35, 36], decreased by about 66% in KO spMNs, whereas the expression of *DCC*, encoding for the receptor of NETRIN-1 with outgrowth-promoting activity [37], was reduced by about 80% (Fig. 6A). Moreover, the expression of *SEMA3E* and *SEMA6D*, belonging to the family of Semaphorins that instruct axon guidance in the developing nervous system [38], was reduced in KO spMNs by about 66% and 46%, respectively. These data imply that *nHOTAIRM1* regulates the activity of several genes crucially implicated in the assembly of neurite networks.

Next, we compared the morphology of both the KO#1 and KO#2 spMNs with that of WT spMNs to verify whether a defective branching phenotype mirrored these molecular analyses. Images of neurite networks composed of WT or KO spMNs (Fig. 6B) were analyzed as described in ref. [39] (Supplementary Fig. 10). Starting from an equal number of cells among all the samples (Fig. 6C), we counted the total number of branches (Fig. 6D) and junctions (Fig. 6E) and measured both the total branch length (Fig. 6F) and the average branch length (Fig. 6G). A statistically significant reduction in the counts of both branch and junction number was found in the two KO neurite networks compared to WT. A lower total branch length was observed in the KO samples in line with these observations. No significant difference in these parameters was estimated between the two KO samples as control. On the contrary, the average branch length was higher in the KO samples compared to WT, as the KO neurite network’s lower density resulted in fewer intersections and, therefore, in longer slab segments. Collectively, these results reveal that *nHOTAIRM1* is necessary for proper neurite outgrowth in spMNs.

### *nHOTAIRM1* controls genes involved in synaptic transmission

**Fig. 7:**
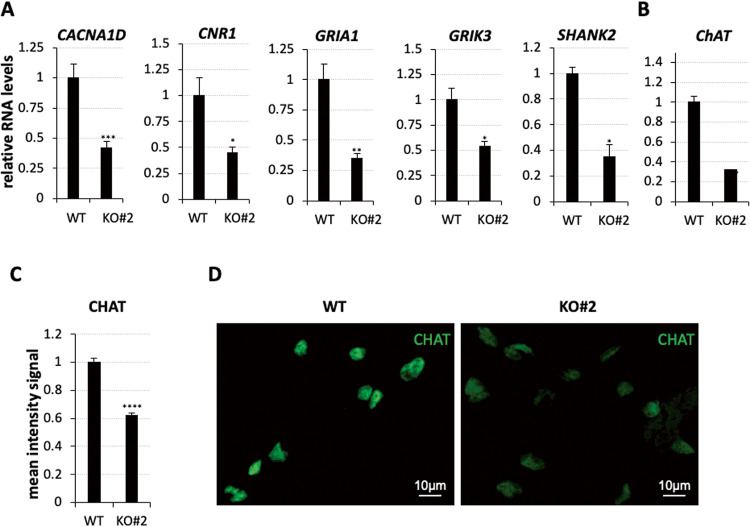
Analysis of synaptic genes in WT and *nHOTAIRM1* KO post-mitotic spMNs. **A** qRT-PCR analysis of synaptic genes (indicated above each panel) in WT and *nHOTAIRM1* KO (KO#2 clone) spMNs (D12). Data (means ± SEM) are expressed in arbitrary units, relative to *ATP5O* mRNA levels, as internal control. For each target, the expression in WT spMNs was set as 1. *N* = 3 biological replicates; *P ≤ 0.05, **P ≤ 0.01, ***P ≤ 0.001, two-tailed Student’s *t* test. **B** qRT-PCR analysis of *ChAT* in WT and *nHOTAIRM1* KO (KO#2 clone) spMNs (D12). Data (means ± SEM) are expressed in arbitrary units, relative to *ATP5O* mRNA levels, used as internal control. The expression in WT spMNs was set as 1. *N* = 3 biological replicates; ***P ≤ 0.001, two-tailed Student’s *t* test. **C**, **D** Quantification of the intensity signal of CHAT protein upon immunostaining in WT and *nHOTAIRM1* KO (KO#2 clone) spMNs (D12). For each sample, the analysis was carried out on 260 cells randomly selected in two different biological replicates. ****P ≤ 0.0001. Unpaired two-tailed Student’s *t* test.

Other genes affected by *nHOTAIRM1* depletion in spMNs were those involved in “synapse organization” and “modulation of chemical synaptic transmission”. They include *CACNA1D*, *CNR1*, *GRIA1*, *GRIK3*, and *SHANK2* genes, all of which were significantly downregulated in KO spMNs (Fig. 7A).

The expression of *CACNA1D* gene encoding for Cav1.3, a member of voltage‐gated L‐type Ca2+ channels essential for synapse maturation and pruning, declined by about 60% in KO spMNs [40–42], whereas the expression of *CNR1* gene, regulating synaptic transmission [43], dropped down by about 56%. *GRIA1*, responsible for MN dendritic architecture [44], and *GRIK3*, relevant in synaptic plasticity and potentiation [45], decreased by about 66% and 47%, respectively, in KO spMNs. Finally, the expression of *SHANK2*, which affects the synaptic connectivity [46], was reduced by about 64%. Besides the aforementioned genes, we also focused on *ChAT*, a gene encoding for choline acetyltransferase, the rate-limiting enzyme in the synthesis of acetylcholine [47], essential for neurotransmission in MNs [48]. We evaluated its mRNA levels via qRT-PCR (Fig. 7B) and protein levels via IF (Fig. 7C, D) in D12 KO *vs* WT spMNs. We found that their levels were significantly reduced when *nHOTAIRM1* was depleted, pointing to a role for the lncRNA as a regulator of synaptic activity in spMNs.

### *nHOTAIRM1* establishes RNA-RNA interactions with a subset of target mRNAs

Being predominantly cytoplasmic, we asked how *nHOTAIRM1* could exert post-transcriptional control of its target genes. A typical role for the cytoplasmic lncRNAs is the competing endogenous RNA (ceRNA) involved in microRNA sponge activity [49]. No data on microRNA-*nHOTAIRM1* interactions in neuronal systems were reported by the LncBase database (https://diana.e-ce.uth.gr/lncbasev3) Therefore, we investigated the interaction between the lncRNA and Argonaute 2 (AGO2), a major component of the miRNA-induced silencing complex (miRISC) [50]. In silico analyses through the catRAPID algorithm (http://s.tartaglialab.com/page/catrapid_group), that predicts protein partners of any given transcript, did not reveal binding propensity of *nHOTAIRM1* for AGO2 protein. Accordingly, AGO2 was not retrieved from *nHOTAIRM1* RAP-Mass Spectrometry experiments, previously performed to identify its protein interactors in spMNs [12]. A cross-linking immunoprecipitation (CLIP) assay, carried out from cytoplasmic extracts of differentiating neurons, further excluded the occurrence of direct interactions between *nHOTAIRM1* and AGO2 (Supplementary Fig. 11). Together, all these data indicate that *nHOTAIRM1* does have the pre-requisites to perform as ceRNA in the tested neuronal context.

**Fig. 8:**
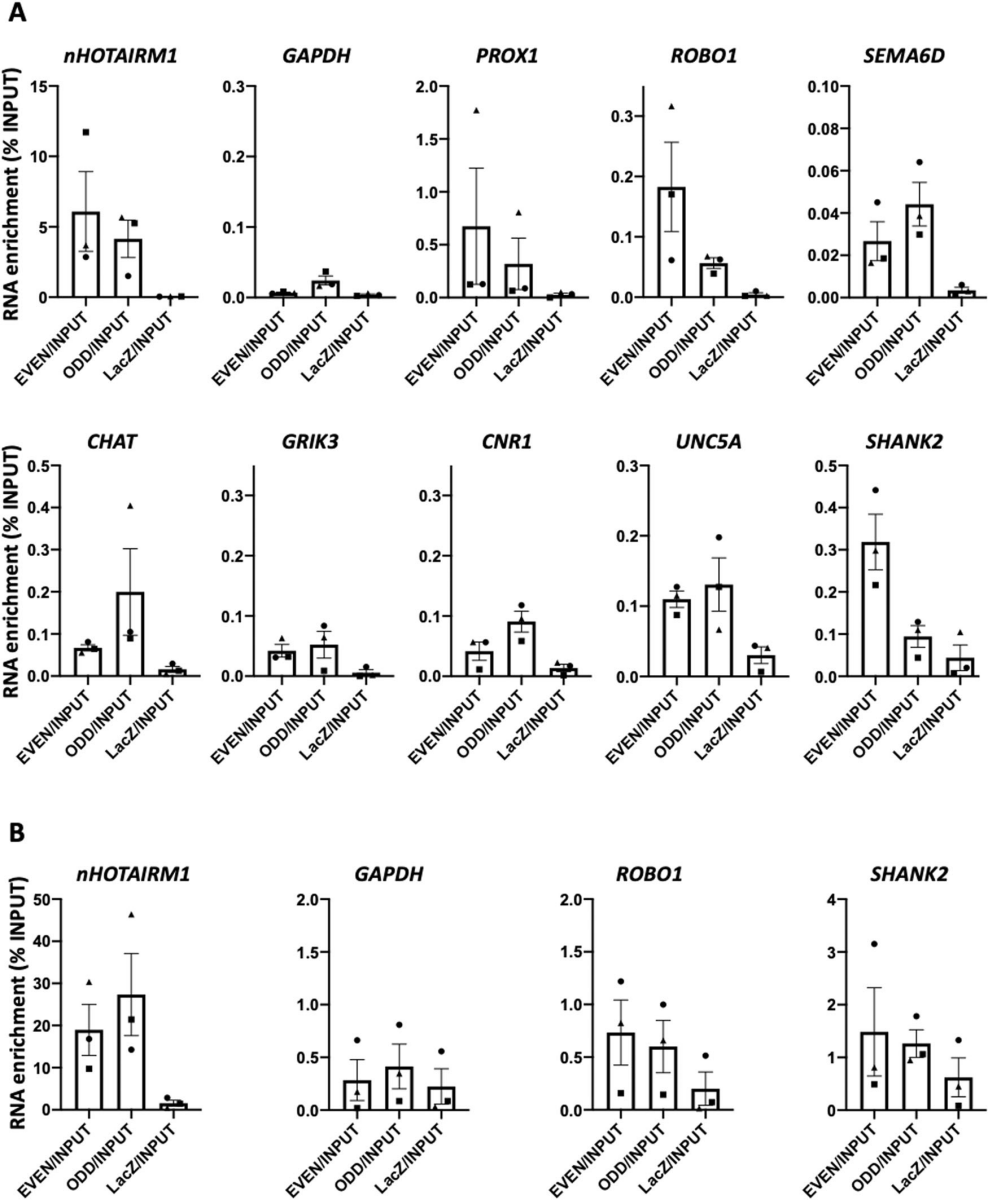
Identification of *nHOTAIRM1* RNA interactors in post-mitotic spMNs. **A** qRT-PCR analysis of *nHOTAIRM1* RNA pull-down experiments performed in native conditions in WT spMN (D12) cytoplasmic extracts. **B** qRT-PCR analysis of *nHOTAIRM1* AMT-crosslinked RNA pull-down experiments performed in WT spMN (D12) total extracts. In both panels, data (means ± SEM) are expressed as percentage of RNA enrichment relative to Input. Boxplots represent the mean value of *N* = 3 independent biological replicates. Symbols (triangle, square and circle) represent values for each biological replicate.

Another mechanism establishing the functional crosstalk between coding RNAs and ncRNAs is mediated by RNA-RNA interactions [49]. RNA pull-down assays were performed in cytoplasmic extracts of WT D12 spMNs, under native conditions. The pull-down fractions enriched for *nHOTAIRM1* were analyzed by qRT-PCR for the presence of mRNAs downregulated in KO spMNs. Among the tested candidates, the transcripts *PROX1*, *ROBO1*, *SEMA6D*, *ChAT*, *GRIK3*, *CNR1*, *UNC5A*, and *SHANK2* were significantly enriched in the *nHOTAIRM1* pull-down fractions (Fig. 8A). To detect direct lncRNA-mRNA pairings occurring in living cells, 4′-aminomethyl-4,5′,8-trimethylpsoralen (AMT)-crosslinked RNA pull-down assays were performed in spMNs. Two out of the eight *nHOTAIRM1* mRNA targets identified in the native RNA pull-down assays, namely *ROBO1* and *SHANK2*, were identified as direct interactors of the lncRNA (Fig. 8B). Through the IntaRNA algorithm (http://rna.informatik.uni-freiburg.de/) we inspected the regions of interaction between *nHOTAIRM1* and the two mRNAs (Supplementary Fig. 12). Interestingly, the region of *nHOTAIRM1* establishing RNA-RNA interactions encompasses the 5′end of exon 3 (nucleotides 644–689) that includes a G-rich tract, predicted as a putative G-quadruplex forming sequence (Supplementary Fig. 13) according to QGRS mapper tool (https://bioinformatics.ramapo.edu/QGRS/). Computational predictions of *ROBO1* or *SHANK2* regions of interaction with *nHOTAIRM1* performed through IntaRNA showed that they map in the 5’ untranslated regions (5′UTRs) of the two mRNAs, according to Ensembl (https://www.ensembl.org/) (Supplementary Fig. 14). This observation supports the idea that *nHOTAIRM1* might play a role in the control of mRNA stability and/or translatability [51].

Overall, these results emphasize the influence of lncRNA-mRNA crosstalk in the multi-step process leading to the generation of functional spMNs.

## Discussion

This study demonstrates that the lncRNA *nHOTAIRM1* is a novel player in the regulatory network governing spMN production and function. Being specifically expressed in the SC, we explored its function in human iPSC-derived spMNs, where it localizes both in the soma and the neurites (Fig. 1C, D). Applying a reverse genetics approach combined with transcriptomics analyses, RNA pull-down experiments, and RNA FISH/IF assays, we uncovered the broad-spectrum regulatory action of *nHOTAIRM1*.

The identification in functional spMNs of *nHOTAIRM1* target genes, whose expression is significantly altered when the lncRNA is depleted, proved that *nHOTAIRM1* participates in (i) spMN differentiation at the expense of IN fate, (ii) neurite outgrowth, and (iii) modulation of synaptic transmission.

Notably, the depletion of *nHOTAIRM1* causes a strong downregulation of several differentiation genes, among which *HB9* and its regulator OLIG2, which are key to triggering MN specification and repressing IN commitment. Accordingly, the absence of the lncRNA during the conversion of iPSCs into spMNs causes a dramatic reduction of specific MN markers, such as *ChAT* [30] and *ISLET1* [ref. [4]] (Fig. 5A) and a concomitant decrease in the number of spMNs, marked by the co-expression of CHAT and ISLET1 proteins (Fig. 5B).

As a corollary to this and similarly to what happens when *HB9* is not functional in mouse-induced MNs [31], the *nHOTAIRM1*-dependent reduction of MN markers is accompanied by the induction of specific IN markers (Fig. 5C and Supplementary Fig. 9), pointing to the lncRNA as a pro-MN molecule, hierarchically positioned upstream of the *OLIG2*/*HB9* module, at the crossroad between MN and IN cell fate (Supplementary Fig. 9B).

To include *nHOTAIRM1* more comprehensively in the complex regulatory network underlying neuronal differentiation, its role in controlling the transition between neuronal precursors and differentiating neurons should also be considered. Indeed, we demonstrated that the lncRNA is required to suppress the expression of the master proneural TF Neurogenin 2 (NGN2) and its downstream neurogenic pathway [12], allowing the differentiation to proceed and the acquired neuronal cell identity to be maintained. This regulative axis fits with the role played by *OLIG2* and *NGN2* in SC development. In MNPs, *OLIG2* expression preserves their undifferentiated state by repressing post-mitotic MN genes, such as *HB9*. On the other side, OLIG2 triggers the expression of *NGN2* in a subset of progenitor cells, priming them to become MNs. Therefore, the relative levels of *OLIG2* and *NGN2* govern the cell fate decision which converts MN progenitors to post-mitotic MNs [29]. Acting on both *OLIG2* and *NGN2*, *nHOTAIRM1* may contribute to establish their proper ratio. The resulting overall picture is consistent with the speculation that *nHOTAIRM1* stands at the apex of a sophisticated molecular circuit composed of *OLIG2*, *NGN2* and their targets, which serves as a gate for timing the correct (motor) neuronal gene expression in the differentiating SC.

Regarding the involvement of *nHOTAIRM1* in establishing proper neuronal connections, it was assessed at both the molecular and phenotypic level. The depletion of *nHOTAIRM1* led to a significant downregulation of genes that influence neuronal wiring and targeting (*NrCAM*), axon guidance (*SEMA3E* and *SEMA6D*), and motor axon trajectories out of the SC (*DCC* and *ROBO1*) (Fig. 6A). Phenotypically, the absence of *nHOTAIRM1* resulted in a defective MN morphology (Fig. 6B), with a significant reduction of the number of branches and junctions and of the total branch length (Fig. 6D–F).

Closely related to this aspect is the control exerted by *nHOTAIRM1* on target genes encoding modulators of synaptic activity (Fig. 7A, B), whose dysfunction has been associated with CNS disorders. This consideration suggests a pathophysiological role for *nHOTAIRM1* in spMNs. For example, diminished activity of *ChAT*, observed in the SC of patients with Amyotrophic lateral sclerosis (ALS), was supposed to be implicated in MN loss-of-function [52] (Fig. 7B, C). In line with this hypothesis, we have demonstrated that *nHOTAIRM1* physically interacts with the RNA binding protein FUS, and that *nHOTAIRM1* expression depends on the levels of FUS [12], whose loss-of-function contributes to neuronal dysfunction and ALS neurodegeneration [53]. Based on this evidence, we plan to better unravel the interplay between *nHOTAIRM1* and ALS mutant *FUS* in the future.

Mechanistically, *nHOTAIRM1* establishes RNA-RNA interactions with a number of its target genes. *PROX1*, *SEMA6D, ChAT*, *GRIK3*, *CNR1*, and *UNC5A* mRNAs establish indirect interactions with *nHOTAIRM1* (Fig. 8A), whereas *ROBO1* and *SHANK2* mRNAs directly interact with the lncRNA (Fig. 8B). It is tempting to speculate that the dynamic association of direct and indirect targets with the lncRNA may play a role along spMN differentiation. Since these interactions were detected in the cytoplasm of post-mitotic MNs, we propose *nHOTAIRM1* as a post-transcriptional regulator of spMN gene expression in this context. To the best of our knowledge, this represents the first evidence of a lncRNA-mediated post-transcriptional control in human spMNs, one only example being described earlier in mouse MNs [54]. The prediction that the direct mRNA interactors of *nHOTAIRM1*, *ROBO1* and *SHANK2*, are bound at their 5’UTRs calls for further mechanistic investigations to elucidate whether the lncRNA regulates its target mRNAs at the level of their stability or translation. Intriguingly, RNA-RNA interaction predictions also indicate that *nHOTAIRM1* binding may occur through a putative a rG4 structure. rG4s are known to be involved in the modulation of gene expression, lncRNA function and disease progression [55].

In conclusion, our findings reveal that *nHOTAIRM1*, as a pleiotropic regulator of multiple molecular pathways involved in MN differentiation, arborization and activity, is essential for ensuring the production of functional spMNs. Since its activity impinges on SC cell fate decisions, this study also provides a rationale for future investigations to clarify how *nHOTAIRM1* contributes to the correct balance between neuronal subtypes in the motor circuits responsible for the overall coordination of locomotion.

## Materials and methods

### Cell culture and MN differentiation

Human induced pluripotent stem cells (iPSCs) used in this study (iPSC-NIL) were derived, maintained and induced to differentiate into spMNs following the methods described in [14].

### Generation of *nHOTAIRM1* KO iPSC-NIL lines

sgRNAs (Dataset 2) were designed using Benchling design tool (https://www.benchling.com/) targeting the regions of *HOTAIRM1* locus. The Cas9 double-strand break target regions were selected, considering FANTOM5 transcription start site data in Zenbu (https://fantom.gsc.riken.jp/zenbu/). PX333 plasmid, encoding the WT Cas9 protein, was purchased from Addgene and sgRNAs were ordered as single-stranded DNA probes (Bio-Fab Research – Rome, IT) and cloned as recommended by the Zhang Lab Protocol (https://media.addgene.org/data/plasmids/62/62987/62987attachment_KiOWQSPn2egB.pdf) resulting in a vector identified as PX333-sgRNAs. HR110PA (System Biosciences – Palo Alto, CA, USA) was used as a backbone to create the donor vector (DONOR). A Poly-adenylation sequence (PAS) was cloned into the DONOR followed by a Neomycin resistance cassette using In-Fusion® HD Cloning Plus Kit (Cat. #638910). Two homology arms (HA) HA1 and HA2, with a length of 800 nt were amplified by PCR using iPSC-NIL gDNA (Kapa HiFi, Takara Bio - Saint-Germain-en-Laye, FR). HA1 was cloned upstream of the PAS, and HA2 was cloned downstream of the PAS. iPSCs were transfected on matrigel-coated dishes through the Neon Transfection System (Life Technologies – Carlsbad, CA, USA), using 100 μl tips in R buffer with the following settings: 1200 V, 30 ms, one pulse. The selection was performed in 800 μg/ml G418 for five days. Single *HOTAIRM1* KO clones were amplified and genotyped.

### Cellular fractionation

iPSC-derived spMNs were subject to subcellular fractionation using the Ambion PARIS Kit (AM1921, Life Technologies). After RNA extraction, equal cytoplasmic and nuclear RNA volumes were retro-transcribed and analyzed by qRT-PCR. Normalizations were based on the total amount of RNA.

### Soma/neurite separation

Soma/neurite separation from iPSC-derived spMNs was performed using modified Boyden chambers and assessed by immunostaining of TUBB3 protein as shown in [19]. RNA samples collected from spMN soma and neurites were then analyzed by qRT-PCR. Enrichment of the neuronal projection marker *COL3A1* in the neurite compartment and of *GNG3* in the soma served as control of the proper separation of the two compartments.

### RNA-Seq and gene ontology analyses

TruSeq Stranded mRNA Library Prep Kit (Illumina - San Diego, CA, USA) was used to obtain sequencing libraries from polyA+ RNA extracted from iPSC-derived WT and *nHOTAIRM1* KO spMNs (3 independent biological replicates).

The sequencing reaction produced 100 nucleotide-long paired-end reads and was performed on a Novaseq 6000 sequencing system (Illumina) with a depth of more than 20 M. To remove adapter sequences and low-quality end bases Trim Galore188 (version 0.6.4_dev) software was used; the minimum read length after trimming was set to 20. Alignment to human GRCh38 genome primary assembly was performed using STAR (version 2.7.9a189).

About 85% of the total reads were successfully mapped to the human genome and most of them were aligned to unique locations. The quantMode TranscriptomeSAM option was used to generate alignments translated into transcript coordinates. The RSEM method was used to quantify the expression levels of the transcripts.

Differential expression analysis was performed using the Bioconductor package DESeq2 that fits a negative binomial generalized linear model on each gene. Only genes displaying in at least three samples a minimum absolute expression value of 10 - as suggested by DESeq2 package developers - were retained. Shrinked log fold change values were obtained by the function lfcShrink which considers the largest fold changes that are not due to low counts and uses these to originate a prior distribution. Genes with padj < 0.05 and absolute logFC > 1 were considered differentially expressed and used for further analysis.

GO over-representation analysis (ORA) was performed on significantly upregulated and downregulated genes having padj < 0.05 and absolute logFC > 1 using the WebGestalt R tool on the GO database (http://geneontology.org/).

### RNA–RNA interaction prediction

IntaRNA version 3.3.2178 was used to map the binding regions between *nHOTAIRM1* and the mRNA interactors found in the AMT-crosslinked RNA pull-down experiments. To obtain in silico predictions of RNA-RNA interactions, we filtered out the RNA transcript isoforms that were not expressed in our system from the RNA-Seq results, by looking at the RSEM output isoforms.

Among the expressed splicing isoforms, we considered the TPM expression values in the WT and KO samples in order to select the protein-coding splicing isoforms that were highly expressed in our system. We subsequently retrieved the FASTA files of these transcripts and used IntaRNA with default parameters to predict mRNA targets sites for *nHOTAIRM1*.

### RNA extraction and analysis

Total RNA was extracted by Direct-zol RNA MiniPrep (R2052, Zymo Research – Irvine, CA, USA). For qRT-PCR assays, cDNA was synthetized by Takara PrimeScript RT Reagent Kit (RR037A, Takara Bio). qRT-PCR detection was performed using SensiFAST SYBR Lo-ROX Kit (BIO-94020, Bioline – Memphis, TN, USA) on a 7500 Fast Real-Time PCR (Applied Biosystem - Carlsbad, CA, USA). *ATP5O* mRNA was used as a reference target. For RNA pull-down assays, total RNA was extracted using miRNeasy Micro Kit (Qiagen - Germantown, MD, USA) and cDNA was synthetized by SuperScript VILO cDNA Synthesis Kit (ThermoFisher Scientific - Carlsbad, CA, USA).

### RNA FISH coupled with IF

iPSC-derived MNs were plated on 12 mm diameter coverslips coated with matrigel (hESC-qualified Matrix Corning 354277) and fixed in 4% paraformaldehyde (Electron Microscopy Sciences - Hatfield, PA, USA) for 10 min at RT. A dehydration step was performed with ice-cold ethanol series (50%, 70%, 100%) in order to store cells at −20 °C until use.

*nHOTAIRM1* was detected via RNA FISH with a mix of 18 biotinylated probes (Dataset 2) as described in [56, 57]. MNs were rehydrated by descendent ice-cold ethanol series (100%, 70%, 50%) and permeabilized in a solution of 0,05% Triton X-100 and 2 mM VRC (R3380, Sigma-Aldrich – St. Louis, MO, USA) in DPBS for 5 min. Cells were then washed three times in DPBS before replacing with 2X SSC buffer (3 M NaCl; 0,3 M sodium citrate in nuclease-free water for a 20X stock solution). Five min incubation in SSC was followed by incubation with pre-hybridization buffer (10% deionized formamide, 47671, Sigma-Aldrich,; 2X SSC in nuclease-free water) for 15 min at 37 °C. MNs were then incubated ON at 37 °C in a slide hybridizer machine (ACD HybEZ™ II Hybridization System) with hybridization buffer (10% deionized formamide; 2X SSC; 10% w/v Dextran sulfate, 2 mM VRC in nuclease-free water) complemented with the biotinylated probes at a final concentration of 50 nM each. The following day cells were washed twice with 2X SSC for 5 min first at 37 °C and then at RT. SSC buffer was then discarded, and coverslips were incubated with TN buffer (Tris HCl pH 7.5 1 M; NaCl 5 M in nuclease-free water) at RT for 10 min. Finally, biotinylated oligos were stained incubating with 1:200 diluted 568-conjugated streptavidin (S11226, Invitrogen – Waltham, MO, USA,) in 4% w/v BSA/TN buffer for 1–2 h at RT in a humid box.

When FISH staining was combined with IF, cells were washed twice with TN buffer and once with DPBS for 5 min at RT, and then were incubated for 1 hour at RT with primary antibodies (anti-βTUBIII, T2200, Sigma Aldrich; anti-Map2, 17490-1-AP, Proteintech, Manchester, UK) diluted in 1% w/v BSA/DPBS. Subsequently, samples were washed three times with DPBS for 5 min at RT and incubated for 45 min at RT with secondary antibodies (Goat anti-Mouse 488, A-11029, Invitrogen; Goat anti-rabbit 488, A-11008, Invitrogen; Donkey anti-Rabbit 594, IS-20152-1, Immunological Sciences - Rome, IT) diluted 1:300 in 1% w/v BSA/DPBS. Lastly, cells were washed three times with DPBS for 5 min at RT, nuclei were counterstained with DAPI solution (1ug/ml/PBS, D9542, Sigma Aldrich) for 5 min at RT and coverslips were mounted with Prolong Diamond Mounting Media (P-36961, ThermoFisher Scientific).

### IF

MNs at D12 were cultured on 0.01% poly-L-ornithine/Murine Laminin 20 μg/ml (Sigma Aldrich), fixed in 4% paraformaldehyde (Electron Microscopy Sciences) for 20 min at 4 °C and washed with PBS. Fixed cells were then permeabilized and blocked with 0.2% Triton X-100/3% BSA/PBS for 15 min at RT. Subsequently, cells were incubated ON at 4 °C with anti-CHAT (ab223346 - Abcam, Cambridge, UK) and anti-ISLET1/2 primary antibodies (mouse clone 39.4D5 - DSHB, Iowa City, IA, USA) diluted 1/200 and 1/50 in 3% BSA/PBS solution, respectively. After washing with PBS, cells were incubated for 45 min at RT in a solution containing 1% donkey serum,1% goat serum in PBS, donkey anti-mouse 647 1:300 (A32787, Invitrogen) and goat anti-rabbit 555 1:300 (A32732, Invitrogen). Nuclei were counterstained with DAPI solution (1 µg/ml/PBS, D9542, Sigma Aldrich).

Images were acquired with inverted confocal Olympus IX73 microscope equipped with a Crestoptics X-LIGHT V3 spinning disk system and a Prime BSI Express Scientific CMOS camera, UPlanSApo 60X (NA 1.42) oil objective, and were collected with the MetaMorph software (Molecular Devices - San Jose, CA, USA). The Z-stack confocal microscopy images were taken automatically (200 nm Z-spacing).

Signal intensity analysis for CHAT was performed on Maximum Intensity Projection with ImageJ software, and it was measured for each single cell taken in account. A total of 260 cells randomly selected in two different biological replicates were considered for WT and KO. Cell count of CHAT + /ISLET1+ cells was performed with “Analyze Particle” tool on binary images obtained with automatic threshold on ImageJ.

### Neurite network analysis

Neurite network analysis was performed in iPSC-derived WT, *nHOTAIRM1* KO#1 and KO#2 spMNs as described in [39] with minor modifications. A Fiji-imageJ macro for semi-automated analysis of MN neurite networks was created adapting the workflow described in [39]. Briefly, Z-projections of confocal images of WT and *nHOTAIRM1* KO spMNs stained with the neurite marker Map2 [ref. [58–60]] were generated. To segment somas starting from the DAPI channel, a nuclei mask was generated with the Huang thresholding algorithm and outliers with a radius lower than 20 pxs were removed. A selection was generated starting from the nuclei mask that was then expanded by 5 pxs (1 px = 0.207 mm) using the “Enlarge selection” function of ImageJ, to account for the small cytoplasmic portion typical of mature MNs.

Subsequently, the Map2 channel was duplicated and two different masks were generated. From the first copy, a high-intensity mask was created thresholding with Moments algorithm upon contrast enhancement (saturated pxs = 0.1%) and Gaussian Blur (σ = 2 μm) filtering. This mask accounts for high-intensity parts of the image, including somas, axon hillock and neurite edges. The second copy was used to generate a low-intensity mask, the LoG mask, to account for thinner parts of neurites. To create the LoG mask, the contrast was enhanced (saturated pxs = 0.1%), the LoG filter from the Process > Math ImageJ menu was used and a threshold was applied to the image using Moments algorithm.

The “Image Calculator” function of ImageJ was then used to combine the high intensity mask with the LoG mask and to subtract the nuclei mask to obtain a final neurite mask.

Finally, the neurite mask was skeletonized and the “Analyze skeleton” function was exploited to determine number of branches, junctions, and end-points per image, as well as total branch length and average branch length.

Number of cells per image was manually counted starting from the nuclei masks, taking advantage of the multipoint tool. Samples were imaged on a Nikon Instrument A1 Confocal Laser Microscope equipped with a 1.49 NA ×100 objective (Apo TIRF 100x Oil, Nikon, Tokyo, Japan) and with a 60x objective. Confocal images were collected with NIS-Elements AR software (Nikon, Tokyo, JP): ND Acquisition module was used for multipoint acquisition of Z-stack images (150–175 nm Z-spacing) of 4 mm thickness.

### RNA pull-down

Native RNA pull-down experiments were performed on cytoplasmic extracts of iPSC-derived spMNs (D12) as described in [61]. Biotinylated DNA probes used to pull down *nHOTAIRM1* are listed in Dataset 2.

AMT-crosslinked RNA pull-down experiments were performed as described in [62]. Biotinylated DNA probes used to pull down *nHOTAIRM1* are listed in Dataset 2.

### Cross-linking immunoprecipitation assay

CLIP assay was performed in cytoplasmic extracts of RA-treated SH-SY5Y neuroblastoma cells as described in this work [12].

### Statistical analyses

Histograms show the mean ± SEM of at least three independent biological replicates. *N* is indicated in Figure Legends. Errors were calculated from relative quantities and then opportunely propagated; statistical significance was determined by two-tailed paired or unpaired Student’s *t* test, as indicated in Figure Legends. A *p*-value < 0.05 was considered statistically significant. *P < 0.05, **P < 0.01, ***P < 0.001, ****P < 0.0001.

### Supplementary information

Data Set 1

Data Set 2

Supplementary Information

Reproducibility Checklist

## Data Availability

The data presented in this study are available in GEO, reference number GSE228681. To review GEO accession number GSE228681, please visit and enter the token gxuzcwcmfrsxjof.

## References

[1] Stifani N (2014). Motor neurons and the generation of spinal motor neuron diversity. Front Cell Neurosci.

[2] Chaudhary R, Agarwal V, Rehman M, Kaushik AS, Mishra V (2022). Genetic architecture of motor neuron diseases. J Neurol Sci.

[3] De Santis R, Garone MG, Pagani F, de Turris V, Di Angelantonio S, Rosa A (2018). Direct conversion of human pluripotent stem cells into cranial motor neurons using a piggyBac vector. Stem Cell Res.

[4] Arber S, Han B, Mendelsohn M, Smith M, Jessell TM, Sockanathan S (1999). Requirement for the homeobox gene Hb9 in the consolidation of motor neuron identity. Neuron.

[5] Kaltezioti V, Antoniou D, Stergiopoulos A, Rozani I, Rohrer H, Politis PK (2014). Prox1 regulates Olig2 expression to modulate binary fate decisions in spinal cord neurons. J Neurosci.

[6] Vangoor VR, Gomes-Duarte A, Pasterkamp RJ (2021). Long non-coding RNAs in motor neuron development and disease. J Neurochem.

[7] Wang KC, Chang HY (2011). Molecular Mechanisms of Long Noncoding RNAs. Mol Cell.

[8] Derrien T, Johnson R, Bussotti G, Tanzer A, Djebali S, Tilgner H (2012). The GENCODE v7 catalog of human long noncoding RNAs: Analysis of their gene structure, evolution, and expression. Genome Res.

[9] Briggs JA, Wolvetang EJ, Mattick JS, Rinn JL, Barry G (2015). Mechanisms of long non-coding RNAs in mammalian nervous system development, plasticity, disease, and evolution. Neuron.

[10] Qureshi IA, Mehler MF (2012). Emerging roles of non-coding RNAs in brain evolution, development, plasticity and disease. Nat Rev Neurosci.

[11] Biscarini S, Capauto D, Peruzzi G, Lu L, Colantoni A, Santini T (2018). Characterization of the lncRNA transcriptome in mESC-derived motor neurons: Implications for FUS-ALS. Stem Cell Res.

[12] Rea J, Menci V, Tollis P, Santini T, Armaos A, Garone MG (2020). HOTAIRM1 regulates neuronal differentiation by modulating NEUROGENIN 2 and the downstream neurogenic cascade. Cell Death Dis.

[13] Mazzoni EO, Mahony S, Closser M, Morrison CA, Nedelec S, Williams DJ (2013). Synergistic binding of transcription factors to cell-specific enhancers programs motor neuron identity. Nat Neurosci.

[14] Garone MG, De Turris V, Soloperto A, Brighi C, De Santis R, Pagani F (2019). Conversion of human induced pluripotent stem cells (iPSCs) into functional spinal and cranial motor neurons using piggyBac vectors. J Vis Exp.

[15] Sepehrimanesh M, Ding B (2020). Generation and optimization of highly pure motor neurons from human induced pluripotent stem cells via lentiviral delivery of transcription factors. Am J Physiol - Cell Physiol.

[16] Solomon E, Davis-Anderson K, Hovde B, Micheva-Viteva S, Harris JF, Twary S (2021). Global transcriptome profile of the developmental principles of in vitro iPSC-to-motor neuron differentiation. BMC Mol Cell Biol.

[17] Smith NC, Wilkinson-White LE, Kwan AHY, Trewhella J, Matthews JM (2021). Contrasting DNA-binding behaviour by ISL1 and LHX3 underpins differential gene targeting in neuronal cell specification. J Struct Biol: X.

[18] Ludwik KA, von Kuegelgen N, Chekulaeva M (2019). Genome-wide analysis of RNA and protein localization and local translation in mESC-derived neurons. Methods.

[19] Garone MG, Salerno D, Rosa A (2023). Digital color-coded molecular barcoding reveals dysregulation of common FUS and FMRP targets in soma and neurites of ALS mutant motoneurons. Cell Death Discov.

[20] Ballarino M, Cipriano A, Tita R, Santini T, Desideri F, Morlando M (2018). Deficiency in the nuclear long noncoding RNA Charme causes myogenic defects and heart remodeling in mice. EMBO J.

[21] Harrison SJ, Nishinakamura R, Jones KR, Monaghan AP (2012). Sall1 regulates cortical neurogenesis and laminar fate specification in mice: Implications for neural abnormalities in Townes-Brocks syndrome. DMM Dis Models Mech.

[22] Asprer JST, Lee B, Wu CS, Vadakkan T, Dickinson ME, Lu HC (2011). LMO4 functions as a co-activator of neurogenin 2 in the developing cortex. Development.

[23] Boukhtouche F, Doulazmi M, Frederic F, Dusart I, Brugg B, Mariani J (2006). RORα, a pivotal nuclear receptor for Purkinje neuron survival and differentiation: From development to ageing. Cerebellum.

[24] Richards AB, Scheel TA, Wang K, Henkemeyer M, Kromer LF (2007). EphB1 null mice exhibit neuronal loss in substantia nigra pars reticulata and spontaneous locomotor hyperactivity. Eur J Neurosci.

[25] Zou M, Luo H, Xiang M (2015). Selective neuronal lineages derived from Dll4-expressing progenitors/precursors in the retina and spinal cord. Dev Dyn.

[26] Sharma K, Sheng HZ, Lettieri K, Li H, Karavanov A, Potter S (1998). LIM homeodomain factors Lhx3 and Lhx4 assign subtype identities for motor neurons. Cell.

[27] Kim M, Fontelonga T, Roesener AP, Lee H, Gurung S, Mendonca PRF (2015). Motor neuron cell bodies are actively positioned by Slit/Robo repulsion and Netrin/DCC attraction. Dev Biol.

[28] Thaler J, Harrison K, Sharma K, Lettieri K, Kehrl J, Pfaff SL (1999). Active suppression of interneuron programs within developing motor neurons revealed by analysis of homeodomain factor HB9. Neuron.

[29] Lee SK, Lee B, Ruiz EC, Pfaff SL (2005). Olig2 and Ngn2 function in opposition to modulate gene expression in motor neuron progenitor cells. Genes Dev.

[30] Wu D, Hersh LB (1994). Choline acetyltransferase: celebrating its fiftieth year. J Neurochem.

[31] Sun M, Ralls S, Wu W, Demmerle J, Jiang J, Miller C, et al. Homeobox transcription factor MNX1 is crucial for restraining the expression of pan-neuronal genes in motor neurons. bioRxiv 10.1101/2021.08.07.455331 (2021).

[32] Bellon A, Mann F (2018). Keeping up with advances in axon guidance. Curr Opin Neurobiol.

[33] Petralia RS, Sans N, Wang YX, Wenthold RJ (2005). Ontogeny of postsynaptic density proteins at glutamatergic synapses. Mol Cell Neurosci.

[34] Sun KLW, Correia JP, Kennedy TE (2011). Netrins: versatile extracellular cues with diverse functions. Development.

[35] Brose K, Bland KS, Kuan HW, Arnott D, Henzel W, Goodman CS (1999). Slit proteins bind robo receptors and have an evolutionarily conserved role in repulsive axon guidance. Cell.

[36] Blockus H, Chédotal A (2016). Slit-robo signaling. Dev (Camb).

[37] Baier H, Bonhoeffer F (1994). Attractive axon guidance molecules. Science.

[38] Pasterkamp RJ (2012). Getting neural circuits into shape with semaphorins. Nat Rev Neurosci.

[39] Pani G, De Vos WH, Samari N, de Saint-Georges L, Baatout S, Van Oostveldt P (2014). MorphoNeuroNet: an automated method for dense neurite network analysis. Cytom Part A.

[40] Pinggera A, Striessnig J (2016). Cav1.3 (CACNA1D) L-type Ca2+ channel dysfunction in CNS disorders. J Physiol.

[41] Hirtz JJ, Boesen M, Braun N, Deitmer JW, Kramer F, Lohr C (2011). Cav1.3 calcium channels are required for normal development of the auditory brainstem. J Neurosci.

[42] Hirtz JJ, Braun N, Griesemer D, Hannes C, Janz K, Löhrke S (2012). Synaptic refinement of an inhibitory topographic map in the auditory brainstem requires functional Cav1.3 calcium channels. J Neurosci: Off J Soc Neurosci.

[43] Tao R, Li C, Jaffe AE, Shin JH, Deep-Soboslay A, Yamin R (2020). Cannabinoid receptor CNR1 expression and DNA methylation in human prefrontal cortex, hippocampus and caudate in brain development and schizophrenia. Transl Psychiatry.

[44] Inglis FM, Crockett R, Korada S, Abraham WC, Hollmann M, Kalb RG (2002). The AMPA receptor subunit GluR1 regulates dendritic architecture of motor neurons. J Neurosci.

[45] Takenouchi T, Hashida N, Torii C, Kosaki R, Takahashi T, Kosaki K (2014). 1p34.3 deletion involving GRIK3: further clinical implication of GRIK family glutamate receptors in the pathogenesis of developmental delay. Am J Med Genet, Part A.

[46] Zaslavsky K, Zhang WB, McCready FP, Rodrigues DC, Deneault E, Loo C (2019). SHANK2 mutations associated with autism spectrum disorder cause hyperconnectivity of human neurons. Nat Neurosci.

[47] Friese A, Kaltschmidt JA, Ladle DR, Sigrist M, Jessell TM, Arbera S (2009). Gamma and alpha motor neurons distinguished by expression of transcription factor Err3. Proc Natl Acad Sci USA.

[48] Maretina MA, Valetdinova KR, Tsyganova NA, Egorova AA, Ovechkina VS, Schiöth HB (2022). Identification of specific gene methylation patterns during motor neuron differentiation from spinal muscular atrophy patient-derived iPSC. Gene.

[49] Noh JH, Kim KM, McClusky WG, Abdelmohsen K, Gorospe M. Cytoplasmic functions of long noncoding RNAs. Blackwell Publishing Ltd; 2018. 10.1002/wrna.1471 PMC5963534 29516680

[50] Höck J, Meister G (2008). The Argonaute protein family. Genome Biol.

[51] Jia L, Mao Y, Ji Q, Dersh D, Yewdell JW, Qian SB (2020). Decoding mRNA translatability and stability from the 5′ UTR. Nat Struct Mol Biol.

[52] Oda Y (1999). Choline acetyltransferase: The structure, distribution and pathologic changes in the central nervous system. Pathol Int.

[53] Ishigaki S, Sobue G (2018). Importance of Functional Loss of FUS in FTLD/ALS. Front Mol Biosci.

[54] Carvelli A, Setti A, Desideri F, Galfrè SG, Biscarini S, Santini T (2022). A multifunctional locus controls motor neuron differentiation through short and long noncoding RNAs. EMBO J.

[55] Li F, Zhou J (2023). G-quadruplexes from non-coding RNAs. J Mol Med.

[56] Santini T, Martone J, Ballarino M (2021). Visualization of nuclear and cytoplasmic long noncoding rnas at single-cell level by rna-fish. Methods Mol Biol.

[57] Vautrot V, Aigueperse C, Branlant C, Behm-Ansmant I (2015). Fluorescence in situ hybridization of small non-coding RNAs. Methods Mol Biol.

[58] Cicchetti F, Lacroix S, Cisbani G, Vallières N, Saint-Pierre M, St-Amour I (2014). Mutant huntingtin is present in neuronal grafts in huntington disease patients. Ann Neurol.

[59] Jeon I, Cicchetti F, Cisbani G, Lee S, Li E, Bae J (2016). Human-to-mouse prion-like propagation of mutant huntingtin protein. Acta Neuropathol.

[60] Song N, Fang Y, Zhu H, Liu J, Jiang S, Sun S (2021). Kir6.2 is essential to maintain neurite features by modulating PM20D1-reduced mitochondrial ATP generation. Redox Biol.

[61] Desideri F, D'Ambra E, Laneve P, Ballarino M (2022). Advances in endogenous RNA pull-down: a straightforward dextran sulfate-based method enhancing RNA recovery. Front Mol Biosci.

[62] Rossi F, Beltran M, Damizia M, Grelloni C, Colantoni A, Setti A (2022). Circular RNA ZNF609/CKAP5 mRNA interaction regulates microtubule dynamics and tumorigenicity. Mol Cell.

