# Supplementary material for: The long noncoding RNA nHOTAIRM1 is necessary for differentiation and activity of iPSC-derived spinal motor neurons
Source: Cell Death Dis. 2023 Nov 14;14(11):741. doi: 10.1038/s41419-023-06196-y (PMC10646148; doi:10.1038/s41419-023-06196-y)
Supplement: Supplementary file 3 — Supplementary Information [file 41419_2023_6196_MOESM3_ESM.pdf]

SUPPLEMENTARY INFORMATION

The long noncoding RNA nHOTAIRM1 is necessary for differentiation and activity of iPSC-derived spinal motor neurons

Paolo Tollis, Erika Vitiello, Francesco Migliaccio, Eleonora D'Ambra, Anna Rocchegiani, Maria Giovanna Garone, Irene Bozzoni, Alessandro Rosa, Annamaria Carissimo, Pietro Laneve, Elisa Caffarelli

The following material is available online:

Figure S1: Computational analysis of RNA-seq data from WT and nHOTAIRM1 KO post-mitotic spMNs (file format: PDF)
Figure S2: Gene ontology analysis (biological process) of significantly differentially expressed genes between WT and nHOTAIRM1 KO in post-mitotic spMNs (file format: PDF)
Figure S3: Gene ontology analysis (cellular component) of significantly differentially expressed genes between WT and nHOTAIRM1 KO in post-mitotic spMNs (file format: PDF)
Figure S4: Gene ontology analysis (molecular function) of significantly differentially expressed genes between WT and nHOTAIRM1 KO in post-mitotic spMNs (file format: PDF)
Figure S5: Gene ontology analysis (KEGG pathway) of significantly differentially expressed genes between WT and nHOTAIRM1 KO in post-mitotic spMNs (file format: PDF)
Figure S6: Gene ontology analysis (Reactome pathway) of significantly differentially expressed genes between WT and nHOTAIRM1 KO in post-mitotic spMNs (file format: PDF)
Figure S7: Validation of differentially expressed genes in *nHOTAIRM1* KO *vs* WT post-mitotic spMNs (file format: PDF)
Figure S8: IF analysis of CHAT and ISLET1 in WT and *nHOTAIRM1 KO* post-mitotic spMNs (file format: PDF)
Figure S9: Analysis of IN marker genes (file format: PDF)
Figure S10: Workflow of the neurite network analysis (file format: PDF)
Figure S11: CLIP assay for AGO2 in the cytoplasmic fraction of 10-day RA-treated SH-SY5Y cells (file format: PDF)
Figure S12: IntaRNA: *nHOTAIRM1* and bound mRNAs interaction predictions (file format: PDF)
Figure S13: Identification of a putative G-quadruplex forming sequence in *nHOTAIRM1* RNA binding region (file format: PDF)
Figure S14: 5' untranslated regions (5'UTRs) of ROBO1 and SHANK2 mRNA isoforms according to Ensembl (file format: PDF)
Dataset 1: RNA-Seq Analysis Data (file format: Excel Workbook)
Dataset 2: List of probes and sgRNAs (file format: Excel Workbook)

Supplementary Figure 1

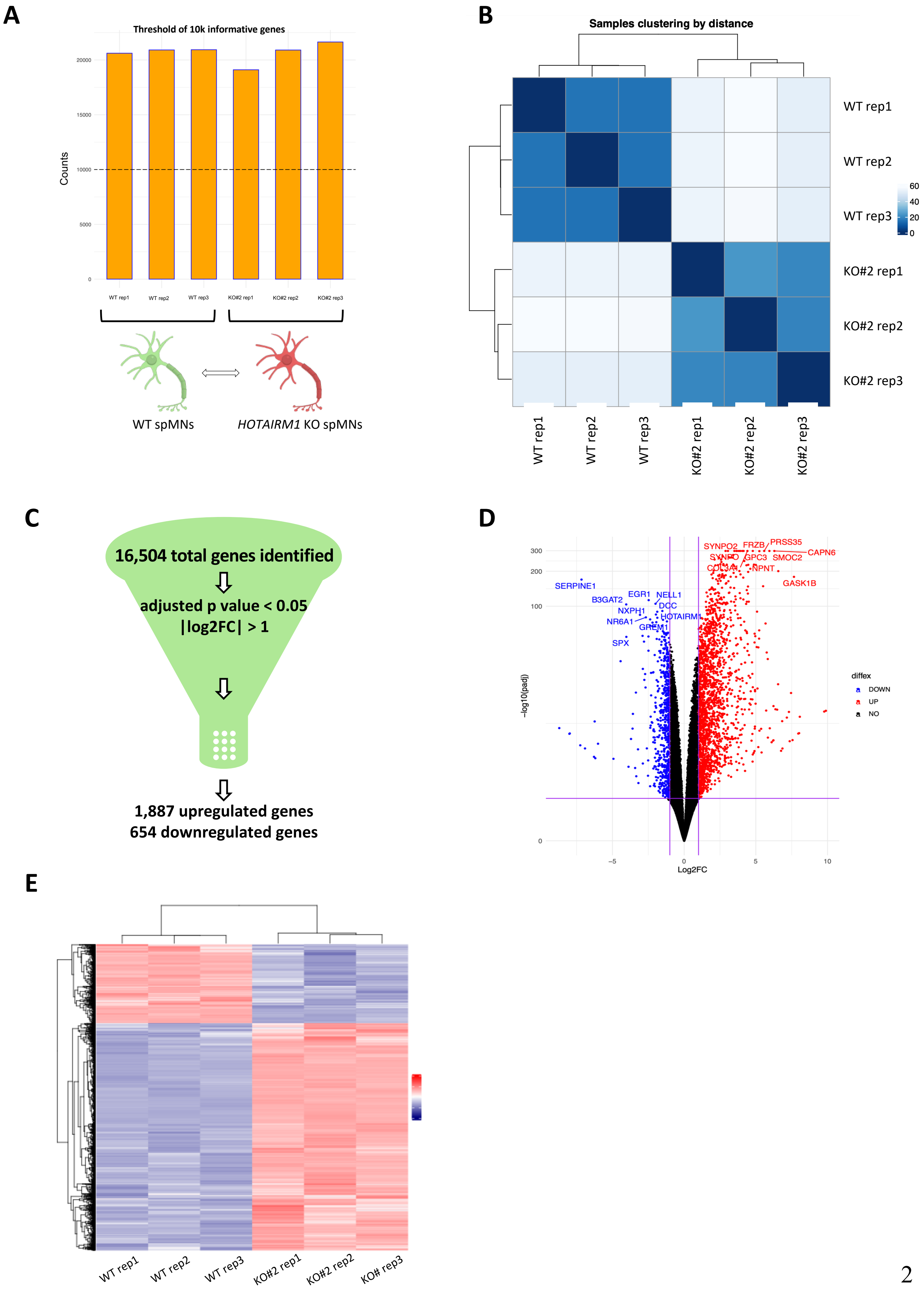

Supplementary Figure 1. Computational analysis of RNA-Seq data from WT and *nHOTAIRM1* KO post-mitotic spMNs

A. The plot reports, for each sequenced sample, the counts of the reads obtained from RNA-Seq analysis.

B. Euclidean distance heatmap showing the similarity in gene expression within the WT and *nHOTAIRM1* KO (KO#2) spMN samples.

C. Graphical visualization of the criteria for the selection of genes significantly differentially expressed upon *nHOTAIRM1* KO.

D. Volcano plot representation of the differentially expressed genes filtered as indicated in panel **C**. The relative difference of expression is represented on the x-axis as the log2 fold change (log2FC) between the KO and WT group of samples. The statistical significance of the differential expression is reported on the y-axis as adjusted p-value (-log10(padj)).

E. RNA-Seq dataset heatmap. Genes are in rows, samples in columns. The expression levels range from blue (low) to red (high).

Supplementary Figure 2

A

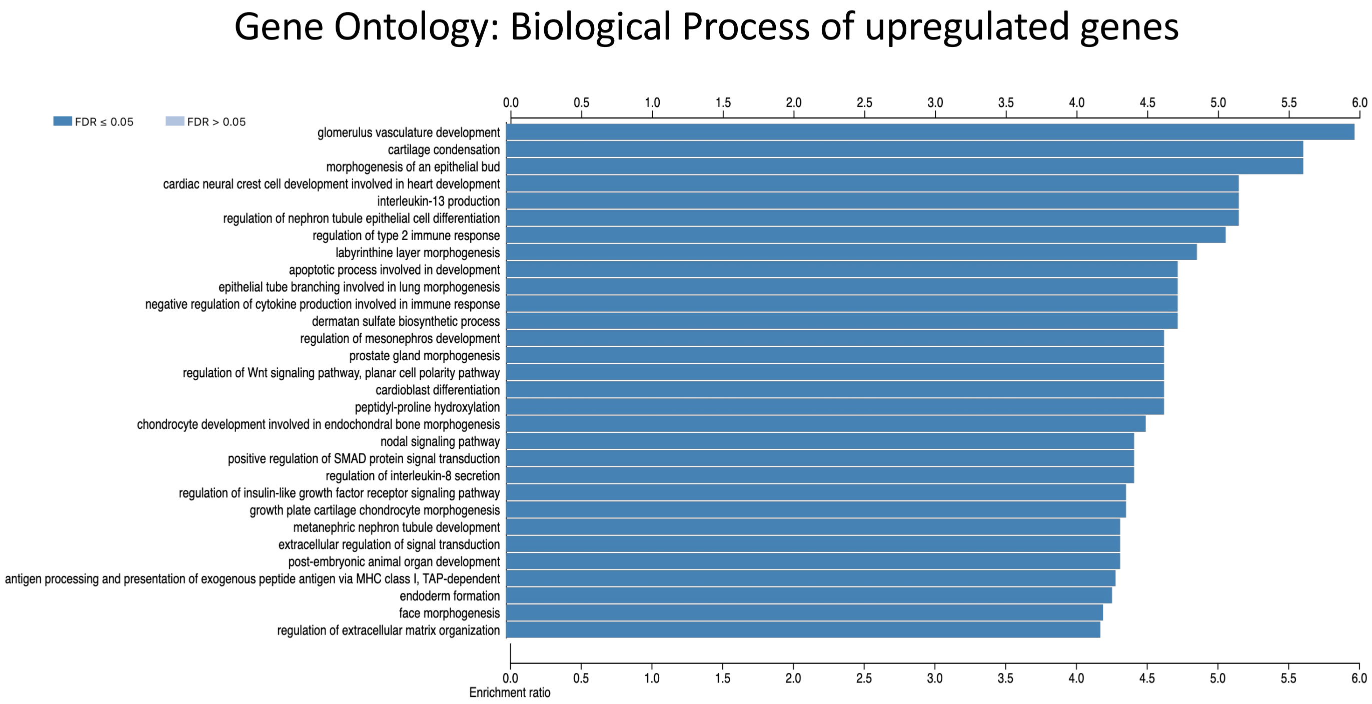

B

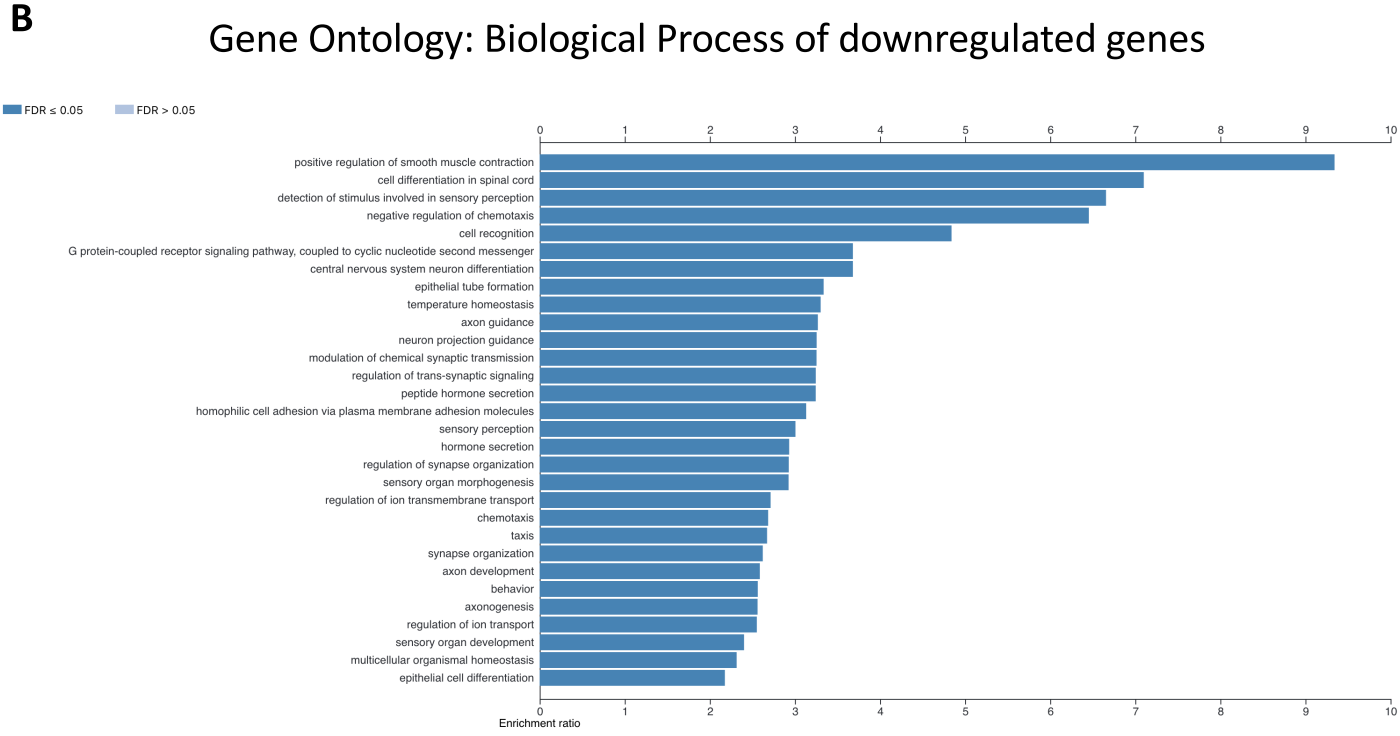

Supplementary Figure 2. Gene ontology analysis of significantly differentially expressed genes between WT and *nHOTAIRM1* KO in post-mitotic spMNs

The plots, produced through the WebGestalt R tool, represent the first 30 biological process categories (indicated on the left) of upregulated (**A**) or downregulated (**B**) genes, with a false discovery rate (FDR) ≤ 0.05, ranked by GO term enrichment ratio.

Supplementary Figure 3

A

Gene Ontology: Cellular Component of upregulated genes

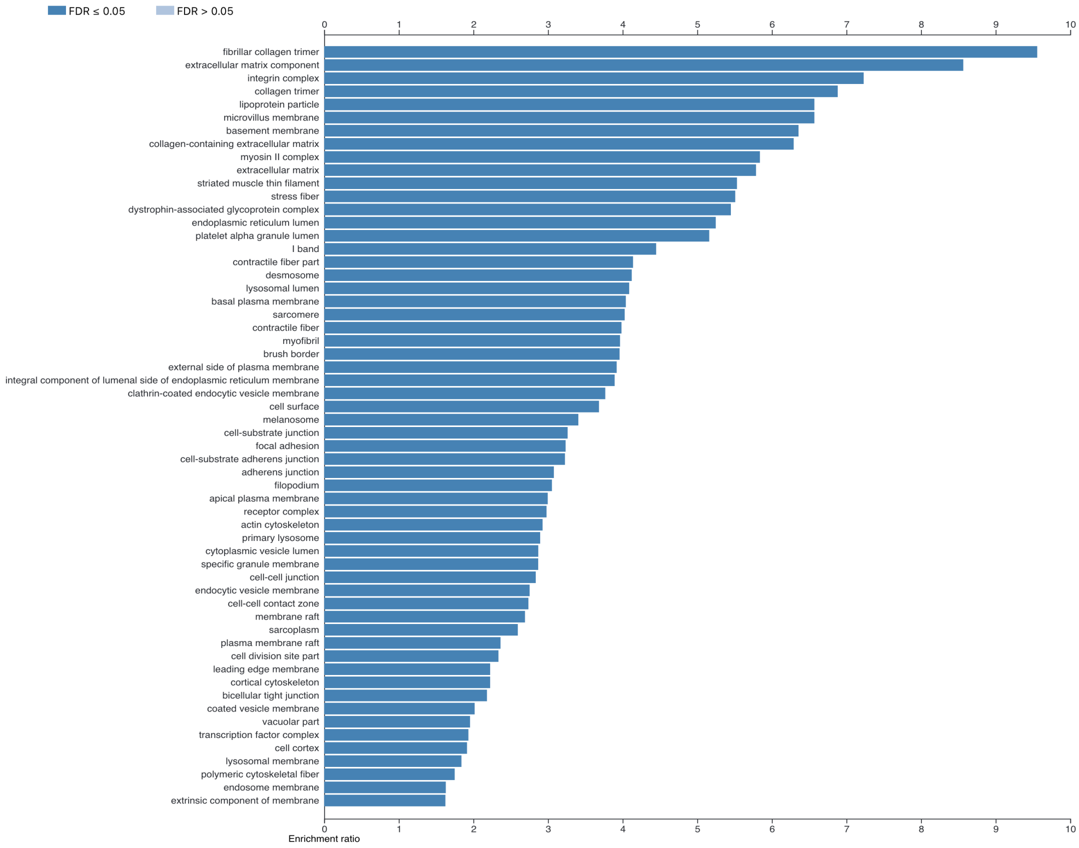

B

Gene Ontology: Cellular Component of downregulated genes

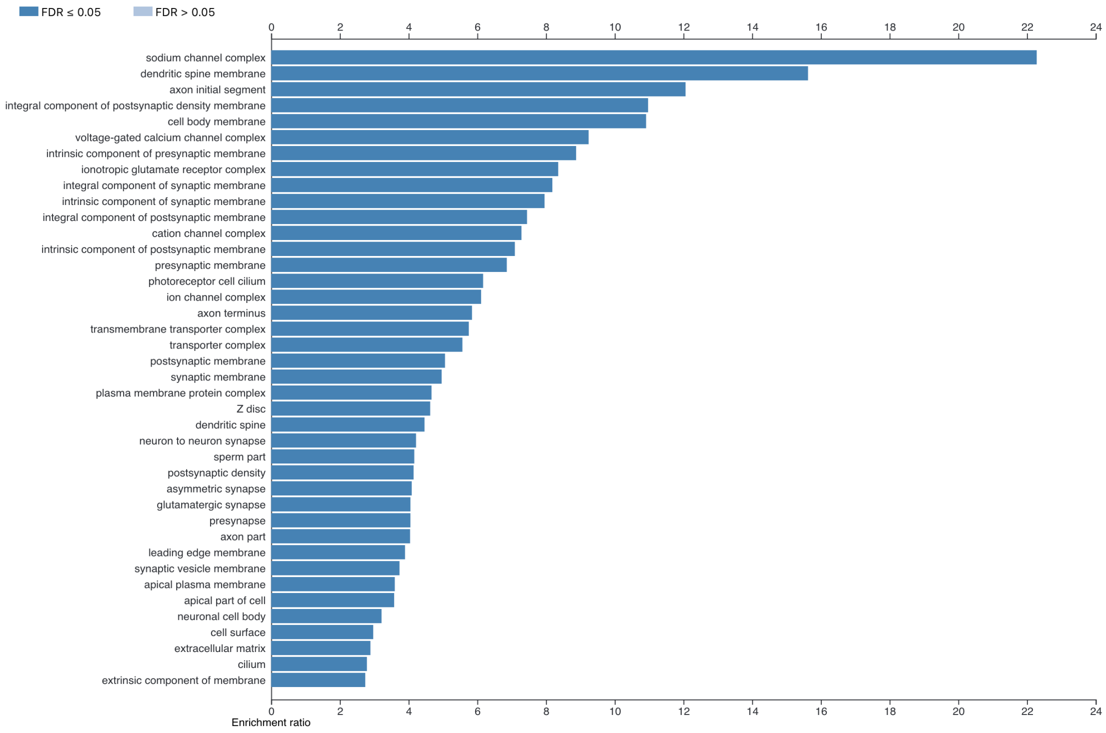

Supplementary Figure 3. Gene ontology analysis of significantly differentially expressed genes between WT and *nHOTAIRM1* KO in post-mitotic spMNs

The plots, produced through the WebGestalt R tool, represent the cellular component categories (indicated on the left) of upregulated (**A**) or downregulated (**B**) genes, with a false discovery rate (FDR) ≤ 0.05, ranked by GO term enrichment ratio.

Supplementary Figure 4

A

Gene Ontology: Molecular Function of upregulated genes

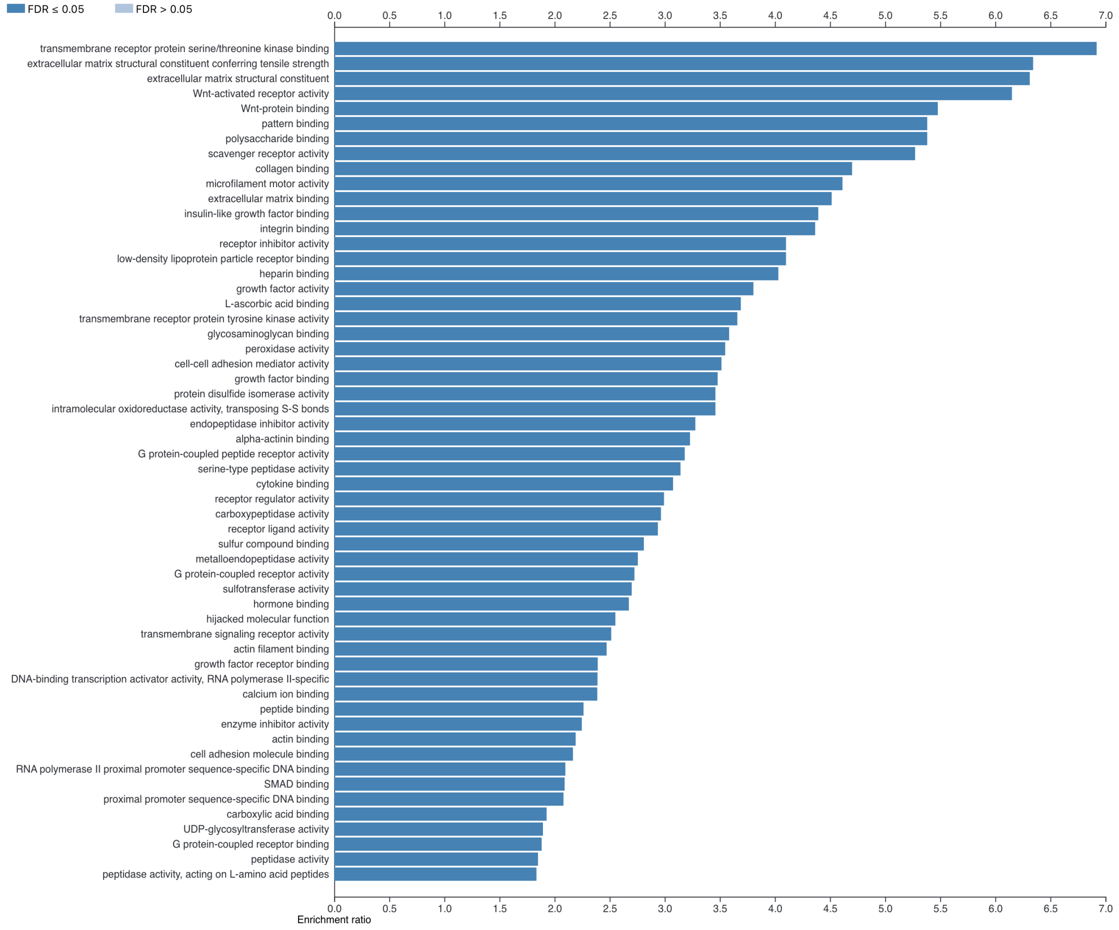

B

Gene Ontology: Molecular Function of downregulated genes

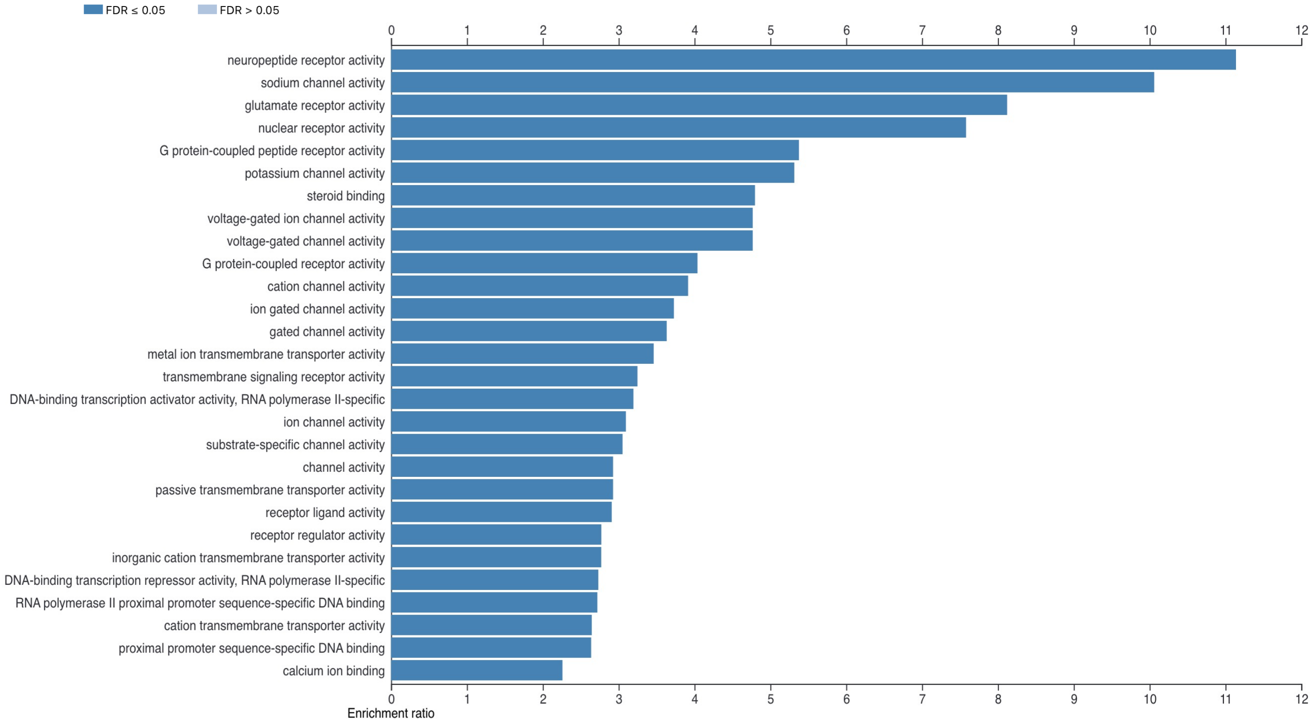

Supplementary Figure 4. Gene ontology analysis of significantly differentially expressed genes between WT and *nHOTAIRM1* KO in post-mitotic spMNs

The plots, produced through the WebGestalt R tool, represent the molecular function categories (indicated on the left) of upregulated (**A**) or downregulated (**B**) genes, with a false discovery rate (FDR) ≤ 0.05, ranked by GO term enrichment ratio.

Supplementary Figure 5

A

Gene Ontology: KEGG Pathway of upregulated genes

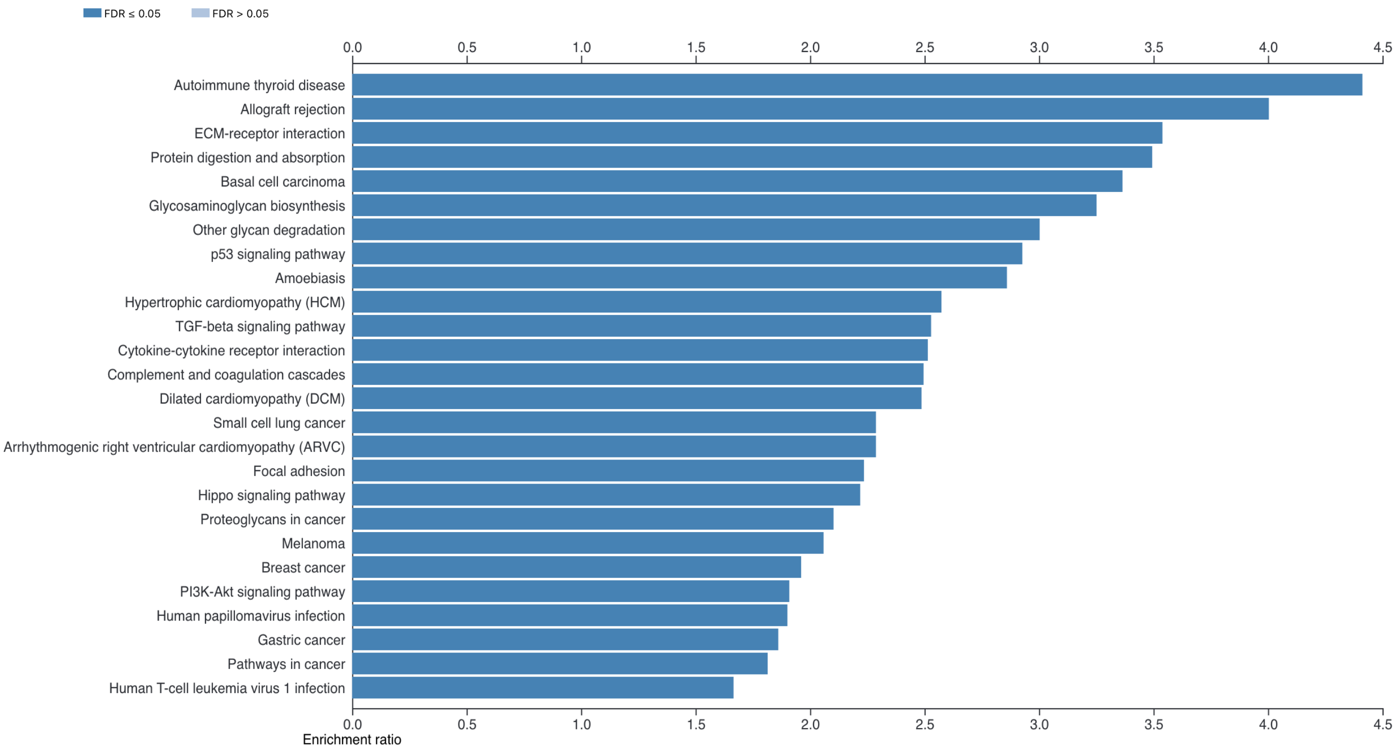

B

Gene Ontology: KEGG Pathway of downregulated genes

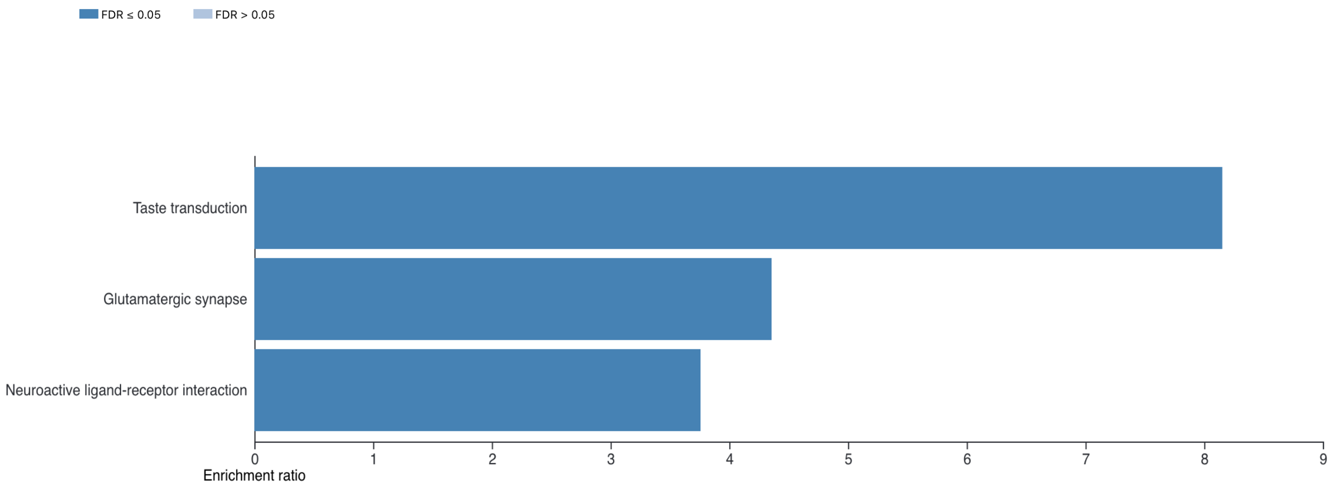

Supplementary Figure 5. Gene ontology analysis of significantly differentially expressed genes between WT and *nHOTAIRM1* KO in post-mitotic spMNs

The plots, produced through the WebGestalt R tool, represent the KEGG Pathway categories (indicated on the left) of upregulated (**A**) or downregulated (**B**) genes, with a false discovery rate (FDR) ≤ 0.05, ranked by GO term enrichment ratio.

Supplementary Figure 6

A

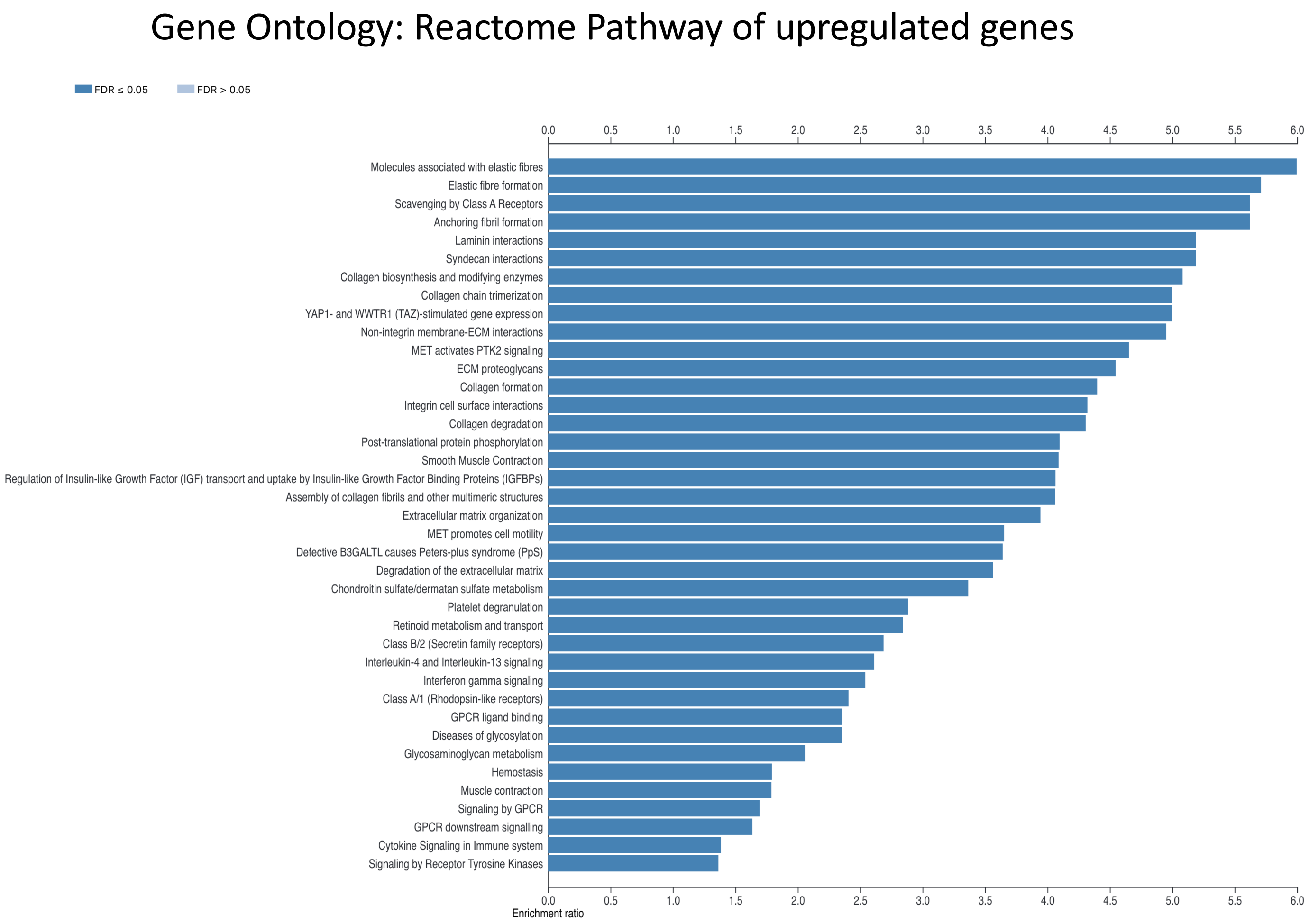

B

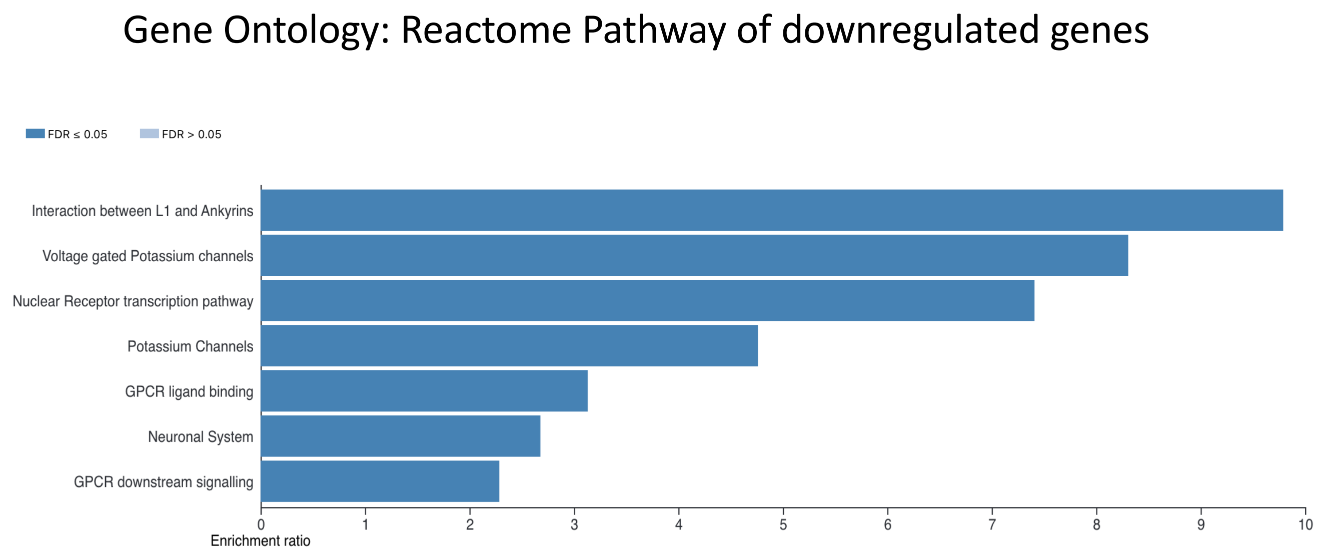

Supplementary Figure 6. Gene ontology analysis of significantly differentially expressed genes between WT and *nHOTAIRM1* KO in post-mitotic spMNs

The plots, produced through the WebGestalt R tool, represent the Reactome Pathway categories (indicated on the left) of upregulated (**A**) or downregulated (**B**) genes, with a false discovery rate (FDR) ≤ 0.05, ranked by GO term enrichment ratio.

Supplementary Figure 7

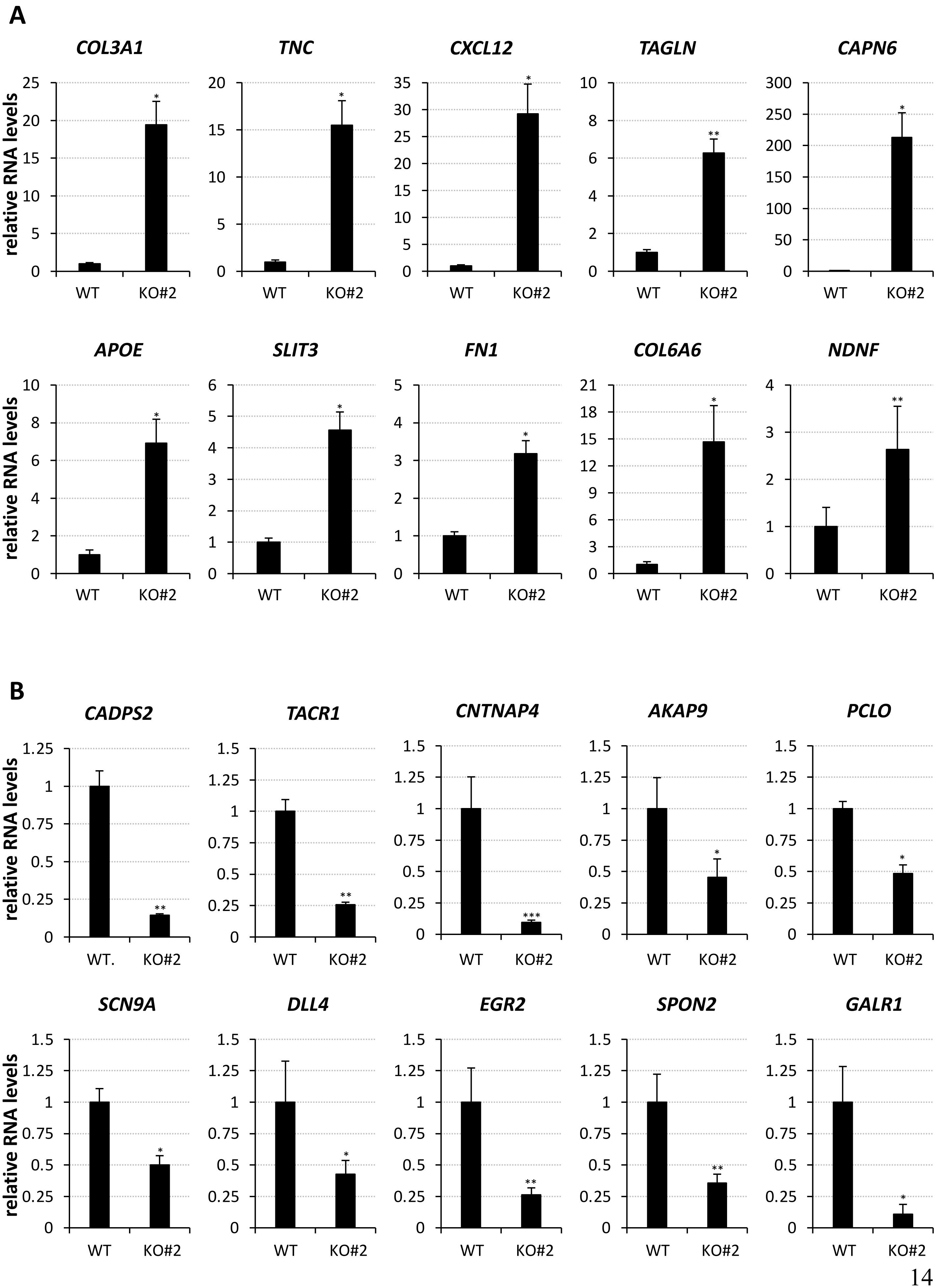

Supplementary Figure 7. Validation of differentially expressed genes in *nHOTAIRM1* KO *vs* WT post-mitotic spMNs

qRT-PCR analysis of upregulated (**A**) and downregulated (**B**) genes in *nHOTAIRM1* KO (KO#2 clone) spMNs (D12) compared to WT. Data (means ± SEM) are expressed in arbitrary units, relative to *ATP5O* mRNA levels, as internal control. For each target, the expression in WT spMNs was set as 1. N=3 biological replicates; *P ≤ 0.05, **P ≤ 0.01, ***P ≤ 0.001, two-tailed Student's t-test.

Supplementary Figure 8

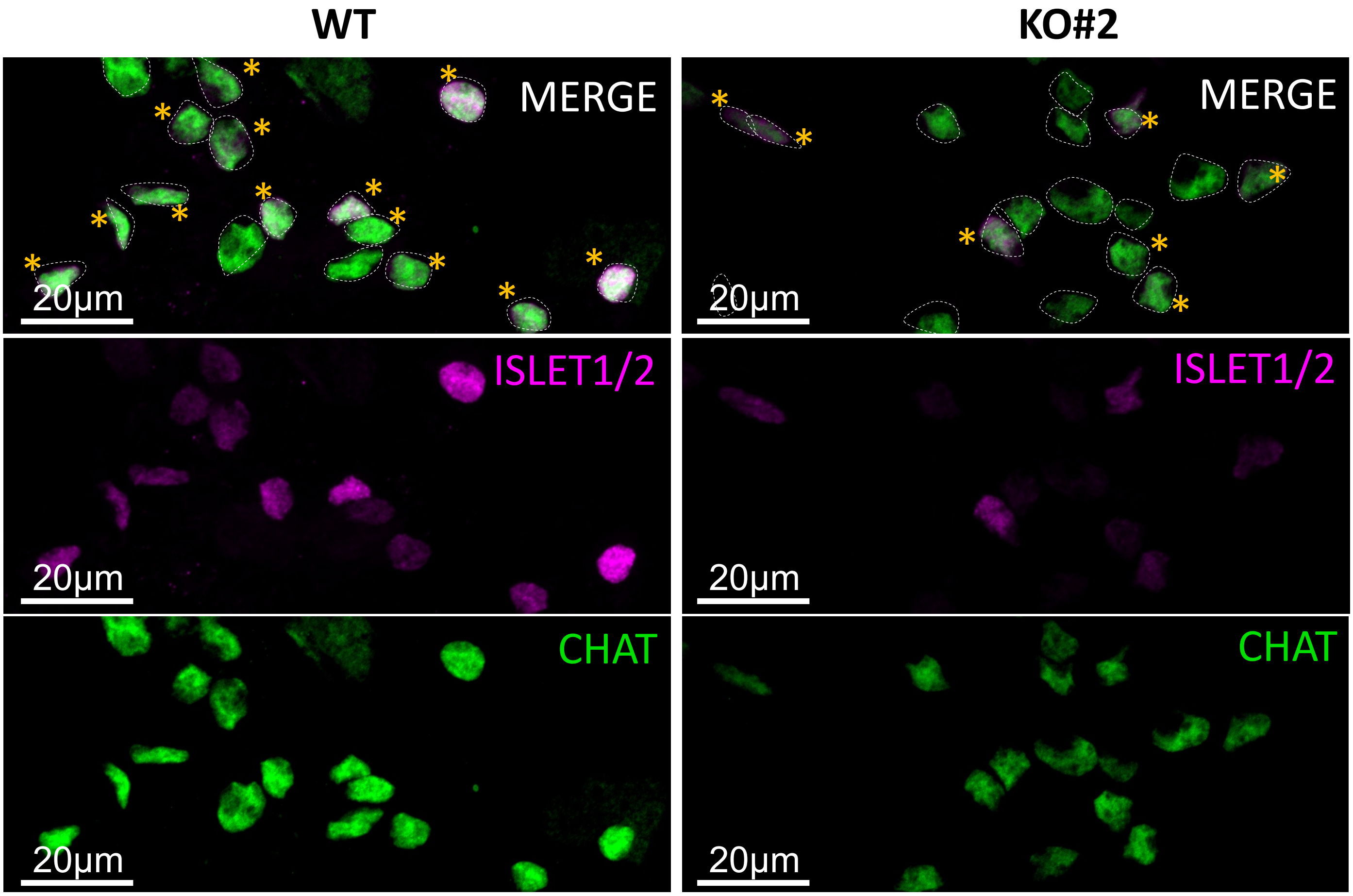

Supplementary Figure 8. IF analysis of CHAT and ISLET1 in WT and *nHOTAIRM1 KO* post-mitotic spMNs

Representative immunostaining fields for CHAT (green) and ISLET1 (magenta) in WT compared to *nHOTAIRM1* KO (KO#2 clone) spMNs (D12). The outline of the nuclei is represented with dotted lines. Yellow asterisks identify cells immunoreactive for both ISLET1 and CHAT. Scale bars correspond to 10 μm.

Supplementary Figure 9

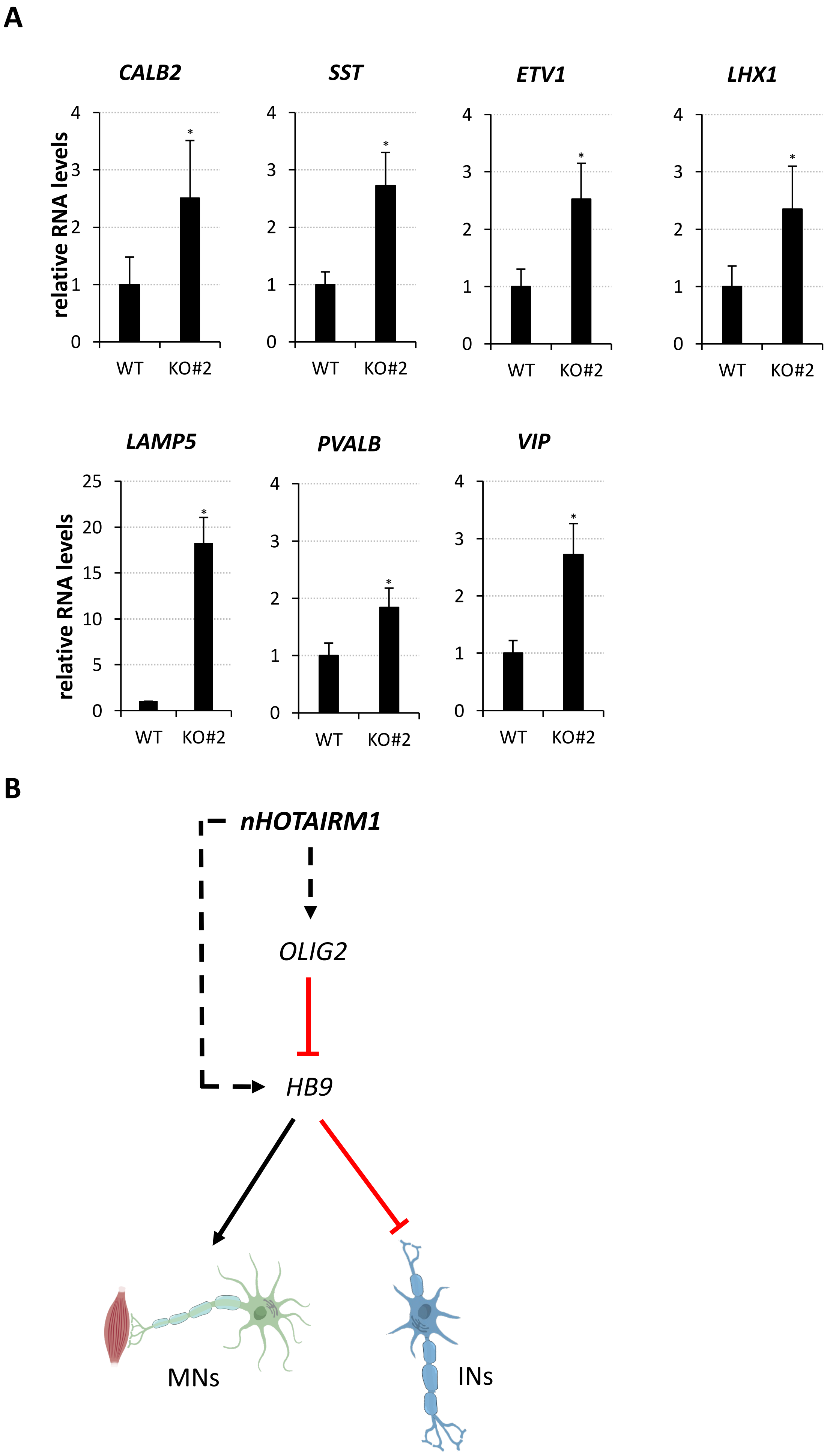

Supplementary Figure 9. Analysis of IN marker genes

A. qRT-PCR analysis of IN marker genes (indicated above each panel) in WT and *nHOTAIRM1* KO (KO#2 clone) spMNs (D12). Data (means ± SEM) are expressed in arbitrary units, relative to *ATP5O* mRNA levels as internal control. For each target, the expression in WT spMNs was set as 1. N=3 biological replicates; $^{*}P \leq 0.05$, two-tailed Student's t-test.

B. Clipart depicts the proposed regulation model in which nHOTAIRM1 is positioned upstream of HB9 and OLIG2 genes, which are key to the binary fate decision between MNs and INs.

Supplementary Figure 10

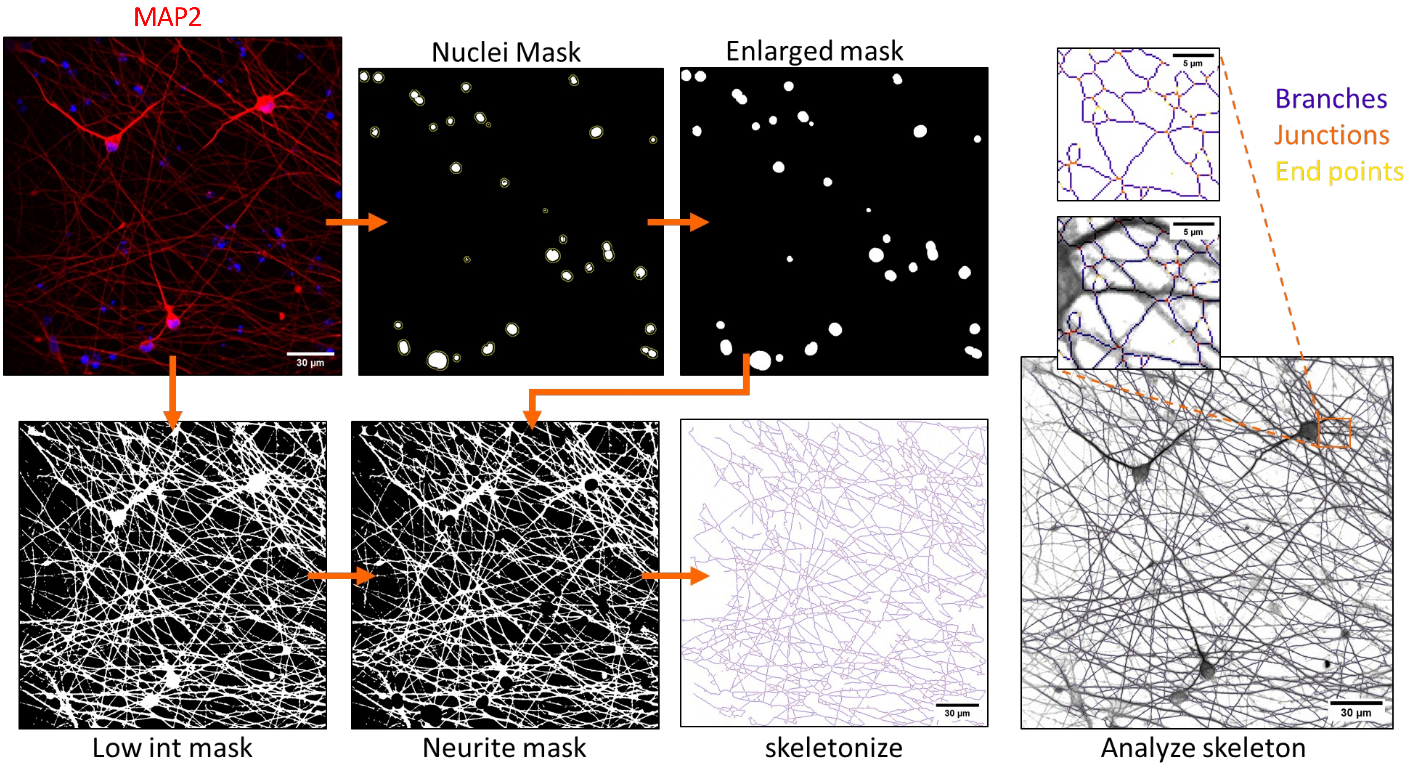

Supplementary Figure 10. Workflow of the neurite network analysis

Representative analysis of WT spMNs. Somata and neurites were visualized by MAP2 staining. An ImageJ custom macro based on the analysis described in Pani et al. 2014 was used to generate *ad hoc* masks (nuclei and neurites) and to skeletonize computed neurite signals. Quantitative analysis was performed by counting or measuring neurite branches, junctions and end-points per acquisition (details in Materials and Methods).

Supplementary Figure 11

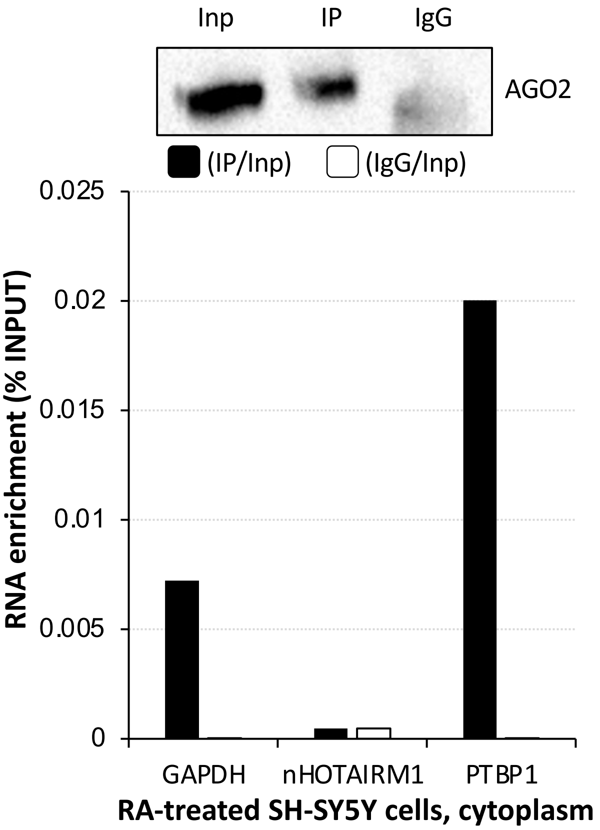

Supplementary Figure 11. CLIP assay for AGO2 in the cytoplasmic fraction of 10-day RA-treated SH-SY5Y cells

Upper panel: immunoblot analysis of AGO2 in Input (Inp) extract, immunoprecipitated (IP) and IgG (IgG) protein fractions. Lower panel: qRT-PCR analysis of RNA enrichment over Input, IP and IgG fractions. Data are expressed as Input percentage. GAPDH was used as a negative control. N = 1.

Supplementary Figure 12

A

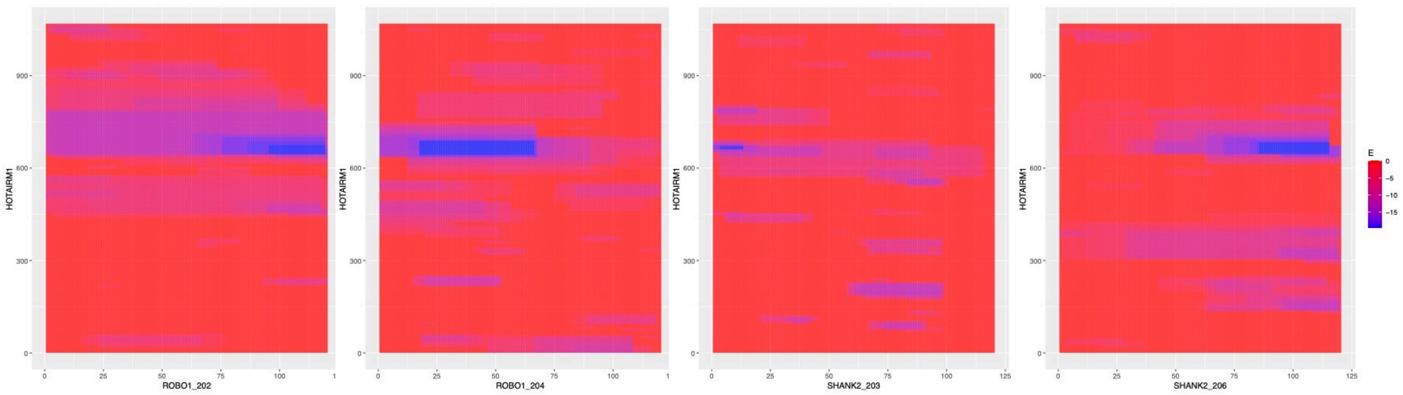

B

```
ENST00000464233.6|ENSG00000169855.20|OTTHUMG00000158843.5|OTTHUMT00000352610.2|ROBO1-202|ROBO1|6927|protein_coding|
             96                            119
              |                             |
5'-GCA...CGAC   -           AU----    -     U-3'
             UUC ACUCUCUCCCU      UUCC CCAC
             ||| +++++++++++      |:|| ||||
             AAG UGGGGGAGGGG      AGGG GGUG
3'-AGA...UGUA   U           GUAUUU    A     UAAA...AAA-5'
              |                             |
            674                            645
HOTAIRM1

interaction energy = -22.07 kcal/mol
```

C

```
ENST00000467549.5|ENSG00000169855.20|OTTHUMG00000158843.5|OTTHUMT00000352613.1|ROBO1-204|ROBO1|4841|protein_coding|
             18                                                 66
              |                                                  |
5'-GUG...UACA      G    -      C           UUGG      A  G A     GGAU...GAU-3'
             UUUGGG ACCC UAUUUC ACUCCCUCCUC    CAUGAG CU U UACA
             ||:::: |||| :||||| +++++++++++    |||:|: |: | :|||
             AAGUUU UGGG GUAAAG UGGGGGAGGGG    GUAUUU GG A GUGU
3'-AGA...AACA      G    U      U           ----      A  G G     AAAA...AAA-5'
              |                                                  |
            689                                                 644
HOTAIRM1

interaction energy = -21.23 kcal/mol
```

D

```
ENST00000409161.5|ENSG00000162105.20|OTTHUMG00000154615.9|OTTHUMT00000259184.2|SHANK2-203|SHANK2|9208|protein_coding|
             4        13
             |         |
       5'-UUA          AAGG...CCG-3'
             AACCCCCUUC
             ++++++++++
             UUGGGGGAGG
3'-AGA...AAAG          GGGU...AAA-5'
             |         |
           671        662
HOTAIRM1

interaction energy = -14.99 kcal/mol
```

E

```
ENST00000424924.5|ENSG00000162105.20|OTTHUMG00000154615.9|OTTHUMT00000259279.2|SHANK2-206|SHANK2|6821|protein_coding|
             86                                     115
              |                                      |
5'-CUG...CGGG    G  CA--        A-     ----- G      AGUCC-3'
             CACC AC    CAGCCCUC  CCCUG      C CUCCA
             |||| ||    ++++++++  |||::      | |||||
             GUGG UG    GUUGGGGG  GGGGU      G GAGGU
3'-AGA...GUUU    G  UAAA        AG     AUUUA G      GUAA...AAA-5'
              |                                      |
            683                                     646
HOTAIRM1

interaction energy = -19.68 kcal/mol
```

Supplementary Figure 12. IntaRNA: *nHOTAIRM1* and bound mRNAs interaction predictions

A. IntaRNA-generated energy maps representing the predicted stability of RNA-RNA interactions between *nHOTAIRM1* and the *ROBO1* or *SHANK2* isoforms expressed in spMNs, according to RNA-Seq. Target mRNA sequences and *nHOTAIRM1* sequence are positioned along the the x- and y-axis, respectively. The free energy of each predicted intermolecular pair ranges from red (higher energy, unstable pairing) to blue (minimal energy, stable pairing).

B-E. IntaRNA *in silico* predictions show: a putative region of interaction between the nucleotides 645-674 of *nHOTAIRM1* and the nucleotides 96-119 of *ROBO1-202* isoform (ENST00000464233.6), with an interaction energy of -22.07 kcal/mol and ii) a region of interaction between the nucleotides 644-689 of *nHOTAIRM1* and the nucleotides 18-66 of *ROBO1-204* isoform (ENST00000467549.5), with an interaction energy of -21.23 kcal/mol.
Regarding *nHOTAIRM1-SHANK2* mRNA direct interaction, IntaRNA predicted: i) a putative region of interaction between the nucleotides 662-671 of *nHOTAIRM1* and the nucleotides 4-13 of *SHANK2-203* isoform (ENST00000409161.5), with an interaction energy of -14.99 kcal/mol and ii) a region of interaction between the nucleotides 646-683 of *nHOTAIRM1* and the nucleotides 86- 115 of *SHANK2-206* isoform (ENST00000424924.5), showing an interaction energy of -19.68 kcal/mol.

Supplementary Figure 13

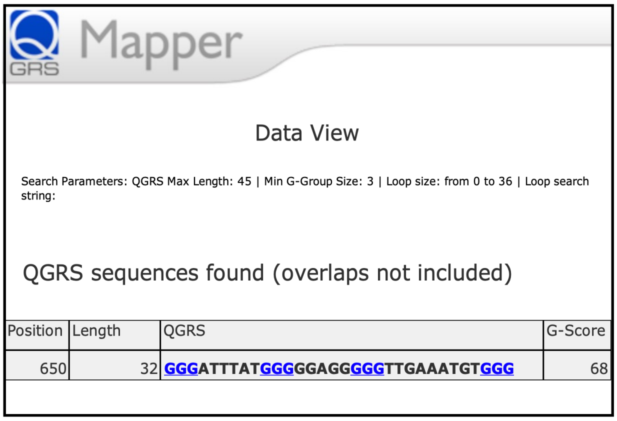

QGRS Mapper

Data View

Search Parameters: QGRS Max Length: 45 | Min G-Group Size: 3 | Loop size: from 0 to 36 | Loop search string:

QGRS sequences found (overlaps not included)

Position	Length	QGRS	G-Score
650	32	GGGATTTATGGGGGAGGGGGTTGAAATGTGGG	68

Supplementary Figure 13. Identification of a putative G-quadruplex forming sequence in nHOTAIRM1 RNA binding region

Putative quadruplex forming G-rich sequence predicted by QGRS Mapper tool on *nHOTAIRM1* RNA sequence. This region is always included in the thermodynamically favored region of lncRNA-mRNA interaction predicted by IntaRNA.

Supplementary Figure 14

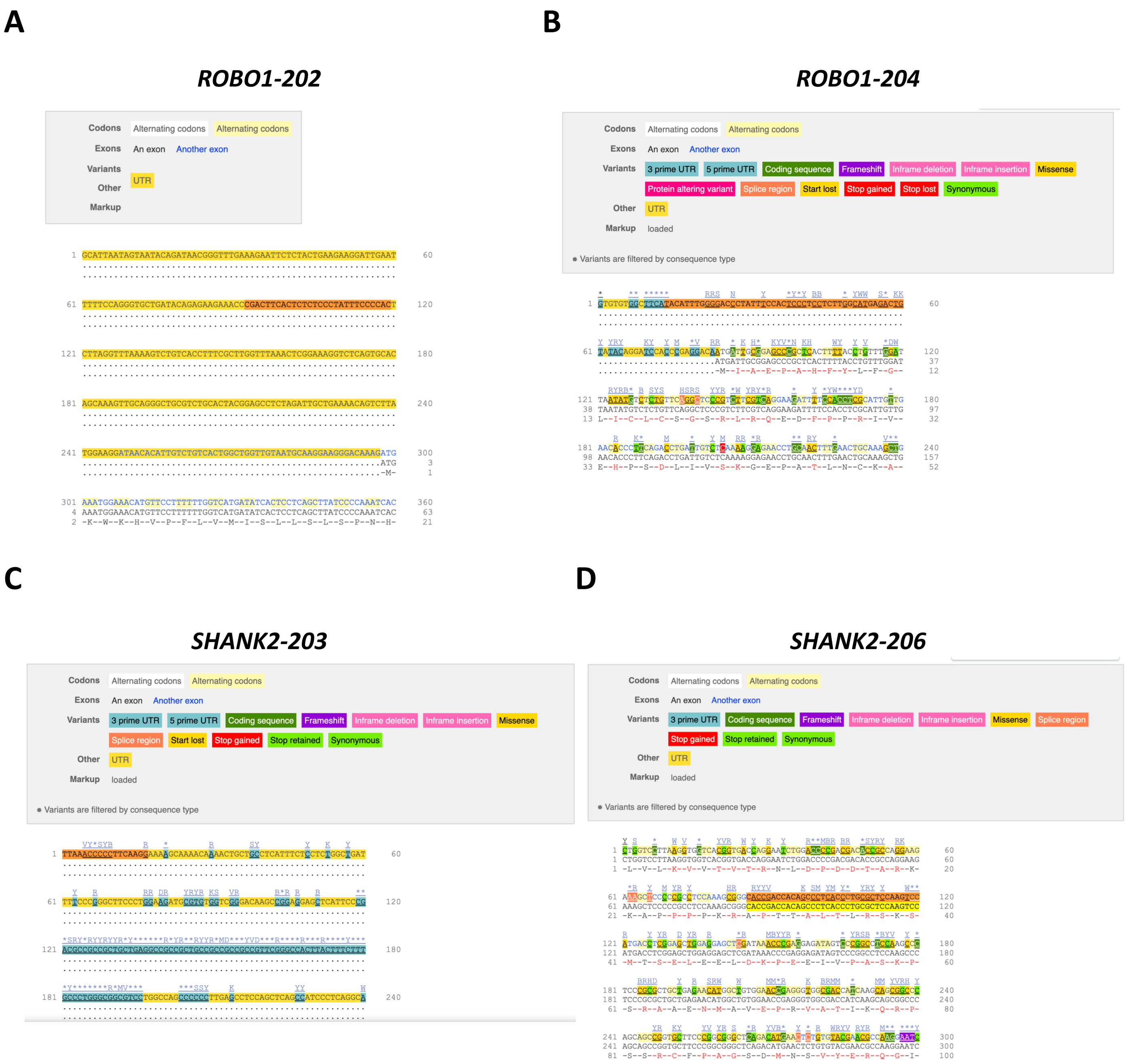

Supplementary Figure 14. 5' untranslated regions (5'UTRs) of ROBO1 and SHANK2 mRNA isoforms according to Ensembl

A-D. Screenshots of sequences and features of the 5' regions of: *ROBO1-202* (**B**), *ROBO1-204* (**C**), *SHANK2-203* (**D**) and *SHANK2-206* (**E**) splicing isoforms according to Ensembl (Release 104, May 2021 used in RNA-Seq analysis). For each mRNA, the sites of interaction with *nHOTAIRM1* (shaded in orange) are located in the 5' UTR.
